# Climate history modulates stress responses of common soil bacteria under experimental drought

**DOI:** 10.1093/ismejo/wraf075

**Published:** 2025-04-18

**Authors:** Nicholas J Bouskill, Stephany S Chacon, Daniela F Cusack, Lee H Dietterich, Liang Chen, Aizah Khurram, Jana Voříšková, Hoi-Ying N Holman

**Affiliations:** Climate and Ecosystem Sciences Division, Lawrence Berkeley National Laboratory, Berkeley, CA 94720, United States; Department of Biological and Ecological Engineering, Oregon State University, Corvallis, OR 97330, United States; Climate and Ecosystem Sciences Division, Lawrence Berkeley National Laboratory, Berkeley, CA 94720, United States; Life and Environmental Science Department, University of California Merced, Merced, CA 95343, United States; Department of Ecosystem Science and Sustainability, Colorado State University, Campus Delivery 1476, Fort Collins, CO 80523, United States; Smithsonian Tropical Research Institute, Apartado Postal 0843-03092, Ancon, Panamá, República de Panamá; Department of Ecosystem Science and Sustainability, Colorado State University, Campus Delivery 1476, Fort Collins, CO 80523, United States; Department of Biology, Haverford College, Haverford, PA 19041, United States; Berkeley Synchrotron Infrared Structural Biology Program, Lawrence Berkeley National Laboratory, Berkeley, CA 94720, United States; Climate and Ecosystem Sciences Division, Lawrence Berkeley National Laboratory, Berkeley, CA 94720, United States; Climate and Ecosystem Sciences Division, Lawrence Berkeley National Laboratory, Berkeley, CA 94720, United States; Laboratory of Environmental Microbiology, Institute of Microbiology, Czech Academy of Sciences, Vídeňská 1083142 20 Prague 4, Czechia; Berkeley Synchrotron Infrared Structural Biology Program, Lawrence Berkeley National Laboratory, Berkeley, CA 94720, United States; Molecular Biophysics and Integrated Bioimaging Division, Lawrence Berkeley National Laboratory, Berkeley, CA 94720, United States

**Keywords:** climate history, real-time metabolic measurements, drought stress, lipid storage, osmolytes

## Abstract

Soil drying challenges microbial viability and survival, with bacteria employing various mechanisms to respond to shifts in osmolarity, including dormancy or metabolic upregulation of osmoprotectants. However, the extent to which these responses are shaped by an organism’s phylogeny, or the climate history of a given environment is poorly understood. This study examines the responses of phylogenetically similar bacteria from semi-arid and humid tropical forest soils to osmotic and matric stress using synchrotron radiation-based Fourier Transform Infrared spectromicroscopy. This non-destructive approach depicts the biochemical phenotype for whole cells under control and stress conditions. We observed that, under osmotic stress, bacteria upregulated cell-signaling pathways, rapidly turned over lipid-storage compounds, and increased osmolyte production. In contrast, matric stress induced a more muted response, typically elevating the production of carbohydrate stress compounds, such as glycine betaine and trehalose. Whereas phylogenetically similar bacteria showed comparable biochemistry under control conditions, climate history played an important role in regulating responses to stress, whereby a stronger metabolic response was observed from semi-arid relative to tropical forest isolates. We conclude that bacterial stress response to drought can be more diverse than previously observed and regulated by both phylogeny and climate history.

## Introduction

Environmental fluctuations—such as changes in pH, temperature, and moisture—can alter the chemical composition of the local environment [1–3], and provoke both short-term (seconds to minutes) and long-term (hours to days) phenotypic and metabolic responses in microbial communities [4]. Soil microorganisms exhibit a range of adaptive traits to protect cellular integrity and maintain metabolism amidst these variable conditions [3, 5]. Moreover, the frequency of environmental changes (occurring on hourly, diel, or seasonal scales), can engender anticipatory responses in individuals and microbial populations that selects for distinct life-history strategies [6–9] and the emergence of seasonally recurring patterns [10].

Soil drying represents a distinct physiological challenge to microbial communities. As soil dries, water forms thin films around soil particles, concentrating aqueous pore water constituents (dissolved nutrients, solutes, toxicants) and limiting the diffusion of substrates and extracellular enzymes [2, 11]. This drying leads to osmotic stress from increasing solute and toxicant concentrations and matric stress from higher surface tension and capillary-bound water, both of which challenge the maintenance of turgor pressure, growth, and cellular viability [12, 13].

Microorganisms exhibit broadly different sensitivities to drought. Gram-negative bacteria appear more sensitive to changing soil moisture compared to Gram-positive bacteria due to differences in cell wall structure [14], which affects the regulation of intracellular osmotic pressure. To mitigate drought stress, bacteria can upregulate pathways to reduce intracellular osmotic potential [15], primarily through the synthesis or accumulation of organic molecules (compatible solutes), like trehalose, and nitrogenous amino acids, such as ectoine, and glycine betaine [16].

Although some common responses of soil bacteria to drought have been described in the literature, a detailed understanding of specific bacterial mechanisms underpinning survival under rapid changes in osmotic potential is lacking. Microbial communities in ecosystems with significant environmental fluctuations are generally less sensitive to stochastic disturbances compared to those in more stable environments (see [17] for greater discussion), and the legacy of drought and extent of soil drying within a given region can all influence the ecological niche of an organism, the traits expressed under stress, and the response to perturbation [18, 19]. This raises the question of whether climate history and drought legacy drive microbial stress response. If so, microorganisms from similar environments should display comparable metabolic responses to stress. Conversely, if microbial responses to stress are hardwired through phylogenetic conservation of functional traits [20], then any responses to stress should be similar for phylogenetically-related organisms from different ecosystems.

We address these broad questions by characterizing the metabolic responses to drought of four common soil bacteria—two Gram-positive, *Arthrobacter pascens* and *Bacillus thuringiensis,* and two Gram-negative bacteria, *Enterobacter asburiae* and *Sphingomonas paucimobilis—*each isolated from soils with different climate histories (semi-arid montane and humid tropical forest soil). We categorize drought stress into the constituents of matric and osmotic stress, each with mild and severe treatments, which may impose diverse metabolic costs on bacterial cells. We further consider whether pre-conditioning cells, through exposure to a lower stress, reshapes metabolic response to more severe stress as noted previously for bacterial populations [19].

We used an ultra-sensitive synchrotron radiation-based Fourier Transform Infrared (SR-FTIR) spectromicroscopy to non-destructively measure changes in cellular components (e.g. water, lipids, proteins, and carbohydrates) and molecular structure originating from exposure to disturbance. Infrared (IR) absorption spectroscopy in the mid-infrared region (4000–650 cm^−1^ in units of wavenumber or reciprocal centimeters) exploits energy transfer from infrared photons of specific vibrational frequencies to distinct vibrational modes of the covalent bonds present in virtually all organic molecules. A highly beneficial feature of this non-invasive method is that these absorptions only occur at resonant frequencies, providing immediate information about the structure and composition of molecules in a biological sample from its mid-IR absorption spectrum even without a priori knowledge (Supplemental Fig. 1). The SR-FTIR approach can provide a 1000-fold increase in signal-to-noise (relative to an FTIR instrument using a halogen light source), for tracking dynamic cellular events in a 3- to 10-μm diameter area. It has previously tracked exposure events over time in a high-throughput and multiplexed manner and has been used to characterize changes in this biochemical composition within fluctuating environments [21, 22].

We employ SR-FTIR on isolate microcolony experiments to test the following hypotheses. (i) Short-term osmotic or matric stress will elicit distinct biochemical responses regardless of an organism’s phylogeny or climate history. (ii) Climate history will shape the metabolic response to stress, whereby organisms adapted to seasonal drought will show a stronger metabolic response (through elevated osmolyte production) than isolates from humid tropical forest soils.

## Materials and methods

### Sites

Bacteria were isolated from two environments; (i) a semi-arid montane (SAM) site (East River, Colorado, 38.92 N, 106.9487 W), which experiences strong fluctuations in soil moisture, where snowmelt saturated soils give way to summer and fall drought, and osmotic potential can reach as low as −20 MPa prior to monsoon season. (ii) a humid tropical forest (HTF) on Buena-Vista Peninsula, Panama (9.185 N, 79.8266 W), that experiences shorter dry periods than the semi-arid site (~133 days on average), with a more moderate decline in soil moisture. The isolates employed in this study are representative of the dominant phyla within these soils, as shown by recent amplicon sequencing studies [23, 24]. The soil properties from each site are provided in Table 1. At each site, 100 g of shallow soil (top 10 cm) was sampled into sterile Whirl-Pak (WI, USA) bags and transported overnight to LBNL. Bacteria were isolated on various media (including R2A, R2G, DNBG, ISS, and M9), and the 16S rRNA genes sequenced to determine taxonomic identify. The specific methods used are described in the supplemental materials. From the resulting isolate library, we picked four isolates (two Gram-positives and two Gram-negatives) from each site that were phylogenetically similar at the 16S rRNA gene level. For ease of reference, the eight isolates were named from both their broad genus name and the site of isolation (either SAM or HTF). For example, *Arthobacter pascens* from the semi-arid montane site would become Arthobacter-SAM.

**Table 1:** Select edaphic parameters for the SAM site in Colorado and HTF site in Panama.

Climate		Soil order	Mean annual temperature (°C)	Mean annual precipitation (m)	Relative humidity (%)	Bulk density	pH	Total C (%)	Total N (%)
Semi-Arid Montane (SAM) (East River, Colorado)	Continental, subarctic^1^	Inceptisol^3^	1^1^	1^1^^,^^*^	45–65^1^	0.8–0.9^3^	6.7^3^	6^5^	0.20^5^
Humid Tropical Forest (HTF) (Buena Vista, Panama)	Monsoonal^2^	Ultisol^4^	26^2^	2.6^2^	>80^2^	0.6–0.7^4^	5.7^4^	5^4^	0.43^4^

^*^60% of the mean annual precipitation falls as snow at this site. ^1^[25], ^2^[26], ^3^[24], ^4^[23], ^5^[27].

### Growth curves

The sensitivity of isolates to increasing NaCl concentrations (as a proxy for osmotic stress) was initially tested in batch culture. Isolates were grown in minimal media (M9 + 20% glucose) with successively higher NaCl concentrations (2–750 mM), which are converted to solute potential (Ψ_s_ = -iCRT, where *i* is the molecules formed in water (2 for NaCl), C is the molar concentration of NaCl, R is pressure constant (= 0.0831 L·Pa·K^−1^·mole^−1^), and T is the ambient temperature (°K)). Isolate growth (at OD 660 nm) was measured hourly over 72 hours and the specific growth rates were calculated during the exponential phase for each isolate under different NaCl concentrations.

### Preparation of cells for SR-FTIR spectromicroscopy of metabolic responses to osmotic and matric stress

**Figure 1:**
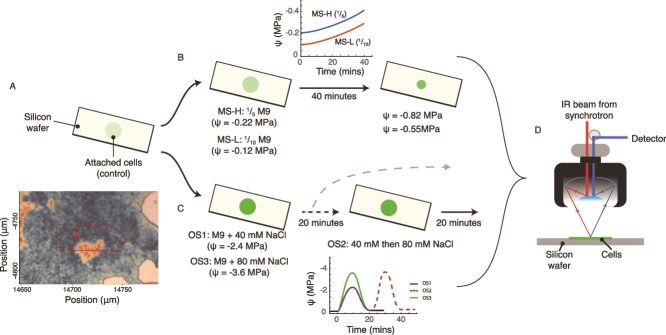
A schematic of the experimental approaches manipulating the water potential for synchrotron-based infrared spectromicroscopy measurements. Details are provided in the materials and methods, however, briefly, panel (A) depicts cell preparation prior to exposure, whereby, a 4 μl aliquot of an exponentially growing axenic cell culture is allowed to adhere to a silicon wafer to form microcolonies (insert), which are either exposed to (B) two different matric stress experiments (MS), where M9 media of different dilution strengths are allowed to evaporate and concentrate the media. Media was either ⅕ strength of initial M9 media (MS-H) or 1/10 strength (MS-L). Isolates were also exposed to (C) three distinct osmotic stress experiments. Two osmotic stress experiments briefly pulse the media with NaCl at a low (40 mM = OS1) and either imaged cell biochemistry afterwards or immediately pulsed the isolates with the high concentration of NaCl (i.e. a sequential step-up experiment of 40 mM to 80 mM = OS2). A final experiment exposed cells to the high (80 mM = OS3) concentration. The phenotypic and metabolic response of the cells to stress is measured using (d) synchrotron-based FTIR, and analyzed through PC-LDA.

Cells were grown to the beginning of the exponential phase in M9 (with 20% glucose) under constant temperature (15°C), before being transferred to ^1^/_100_-strength M9 for attachment to silicon wafers. 4 μl of cells were placed onto a silicon wafer to promote attachment (Fig. 1A). The cells were washed three times in DI water to remove the unattached cells, and the remaining media. Ψ_s_ within this media at room temperature and atmospheric pressure was estimated to be ~21 kPa. Subsequent experiments were designed to manipulate this value (Fig. 1B/C), allowing the biochemical response of microbes to changes in Ψ_s_ to be evaluated by SR-FTIR.

### Matric potential

Soil drying results in the formation of water films on soil particles, reducing diffusion and increasing the matric potential within the film. We replicated this process by allowing a solution of either ^1^/_5_ (termed MS-H) or ^1^/_10_ (MS-L) strength M9 media to dry down to 50% of the original volume of 4 μl (Fig. 1B). We estimated that this would increase Ψ_s_ from −220 kPa to −820 kPa under MS-H, and − 120 kPa to −550 kPa under MS-L. This value is still within the estimated range of microbial activity during soil drying [14].

### Osmotic potential

As soils dry, salts accumulate and present a significant physiological challenge to microbial cells. Osmotic shock under rapid soil drying can significantly impair microbial cell function or provoke a metabolic response. Herein, we examined whether bacteria could mount a metabolic response to an acute osmotic shock by rapidly increasing NaCl in the media through the addition of either 40 mM NaCl (termed OS1, estimated Ψ_s_ ~ −2.4 MPa), or 80 mM NaCl (OS3, estimated Ψ_s_ ~ −3.6 MPa). We further asked whether pre-exposure to a lower concentration of NaCl can condition the cell upon subsequent acute exposure to a higher dose (80 mM). Previous work has shown that an immediate switch between a low and high dose of NaCl can invoke an anticipatory response under the higher dose [19]. This experiment, termed OS2, exposed the attached isolates to a 40 mM NaCl concentration for 20 minutes, before immediately increasing the NaCl concentration to 80 mM for an additional 20 minutes. For all experiments, the cells were washed in DI water and imaged under the FTIR microscope.

### Flow cytometry

Live/dead staining was used to ascertain mortality rates under the OS and MS experiments. The OS and MS experiments described above were replicated in slightly larger volume (10 μl), and live/dead staining carried out following the manufacturers protocol (*BacLight* bacterial viability and counting kit, L34856, Molecular Probes, OR). Briefly, 10 μl control or treatment cells were combined with a 3.34 mM solution of SYTO 9 nucleic acid stain, and a 30 mM solution of propidium iodide, and incubated for 15 minutes at room temperature. This solution was passed through a cell sorter (Attune NxT Flow Cytometer, Thermo Fisher, Waltham, MA) to enumerate the proportion of live/ dead cells post treatment.

### SR-FTIR spectromicroscopy

All SR-FTIR spectromicroscopy measurements were carried out at the infrared beamline 1.4.3 of the Advanced Light Source (Lawrence Berkeley National Laboratory, Berkeley, CA; http://infrared.als.lbl.gov/) following previously published protocols. Briefly, mid-infrared photons emitted from the synchrotron were focused on the monolayers of bacteria on the silicon-coated surface by the all-reflective optics infrared microscope (Fig. 1D) equipped with a 32X, 0.65 numerical aperture objective. Each IR spectrum was raster scanned in the transflection mode using the IR microscope to map the bacterial monolayers at a 5 μm step size using Omnic 7.2 spectra software (Thermo Fisher Scientific, Waltham, MA). Each scan was repeated 64 times over the mid-infrared wavenumber range of 650–4000 cm^−1^, and each scan was recorded at a spectral resolution of 4 cm^−1^ with an adsorption peak position accuracy of ^1^/_100_ cm^−1^. Each SR-FTIR spectrum covered around 20 bacterial cells. All spectra for each sample map were exported for data pre-processing and multivariate analysis.

### SR-FTIR data pre-processing and chemometrics

Only SR-FTIR data in the region spanning 900 to 1800 cm^−1^ were subject to data pre-processing and multivariate analysis. Preliminary work demonstrated this region to be optimal (in terms of specificity and consistency) for distinguishing between different microbial isolates and stress treatments. In-house scripts were used to remove spectra from non-monolayer distributed microbial cells or spectra with a poor signal-to-noise. Spectra that exhibited scattering effects were subject to the Kohler EMS correction algorithm to correct the spectral line shape distortion [28]. All pre-processed spectra were vector normalized and analyzed after mean-centering using principal components analysis followed by linear discriminant analysis (LDA). The resulting PC-LDA score plots permits visual determination of clustering within and between experiments. Similarly, PC-LDA loadings and cluster vector spectra plots (Supplemental Figures) identify the molecular functional groups and chemical bonds underpinning the ordination of different isolates.

The vector-normalized spectra were also transformed to second derivative spectra (7-point Savitzky–Golay smoothing, polynomial order 3) and followed by second derivative spectroscopy analysis. Second derivative spectroscopy analysis can reveal more specific information of adsorption peaks that are either small or poorly resolved in the original spectrum but are likely biochemically important markers. Moreover, the differentiation step in the second derivative spectroscopy can remove baseline effects and increase feasibility of quantitative analysis. The application of the second derivative spectroscopy to perform spectra quantitation was often considered not practical because it could reduce the signal-to-noise ratio in the data by approximately an order of magnitude. However, in the present study, infrared light derived from the synchrotron has a 100 to 1000-fold increase in the signal-to-noise (relative to an FTIR instrument with a conventional infrared light source, [29, 30]).

Given sufficient signal-to-noise and molecular specificity the second derivative can permit quantitative analysis [31, 32]. From the Beer–Lambert Law, the measured absorbance at a frequency, ω, can be expressed as,


(1) \begin{equation*} A\left(\omega \right)=\alpha \left(\omega \right)\mathrm{I}c \end{equation*}

where, A is the frequency-dependent absorbance, α is the frequency-dependent absorption coefficient, Ι is the optical pathlength, and c is the concentration. Differentiating equation 1 twice gives,


(2) \begin{equation*} \frac{d^2A\left(\omega \right)}{{d\omega}^2}=\frac{d^2\alpha \left(\omega \right)}{d{\omega}^2}\mathrm{I}c \end{equation*}

from equation 2 quantitative information can obtained from the second derivative spectra, since Ι and c are independent of the frequencies.

The score plots of PC-LDA were used for a visual representation of the separation of the stress response in microbes from different geographic origins based on their whole metabolic response to stress. To identify how different metabolic responses contribute to discrimination between samples we compared the mean adsorption spectra from the cluster loading plot. We focus our analysis here on the 900–1800 cm^−1^ fingerprint region that provides information on the production, transformation, or depletion of a range of compound classes, including the fatty acid carbonyl region (1680–1770 cm^−1^), protein secondary structure amide I (1600–1700 cm^−1^), amide II (1519–1544 cm^−1^) regions, and carbohydrate (1000–1300 cm^−1^) regions. Spectral interpretations were guided by the second derivative spectra and in-house reference spectra for polysaccharides, proteins, and water vapor. Quantitative identification of specific compounds related to bacterial storage compounds—polyhydroxyalkanoates (PHA), polyhydroxybutyrates (PHB), glycogen, polyphosphate—and metabolic stress compounds—trehalose, ectoine, glycine betaine, proline, glycogen—were identified by comparison with the second derivative spectra specific to each compound (Supplementary Fig. 4). The experimental spectra were analyzed for multiple characteristic peaks representing each compound (Supplementary Table 3), with an identification threshold of 75%. A z-test was performed to identify metabolites that either statistically significantly increase or decrease relative to the baseline (control) spectra.

## Results

### Growth rates and survivorship measurements emphasize the non-lethal nature of the experimental stress

**Figure 2:**
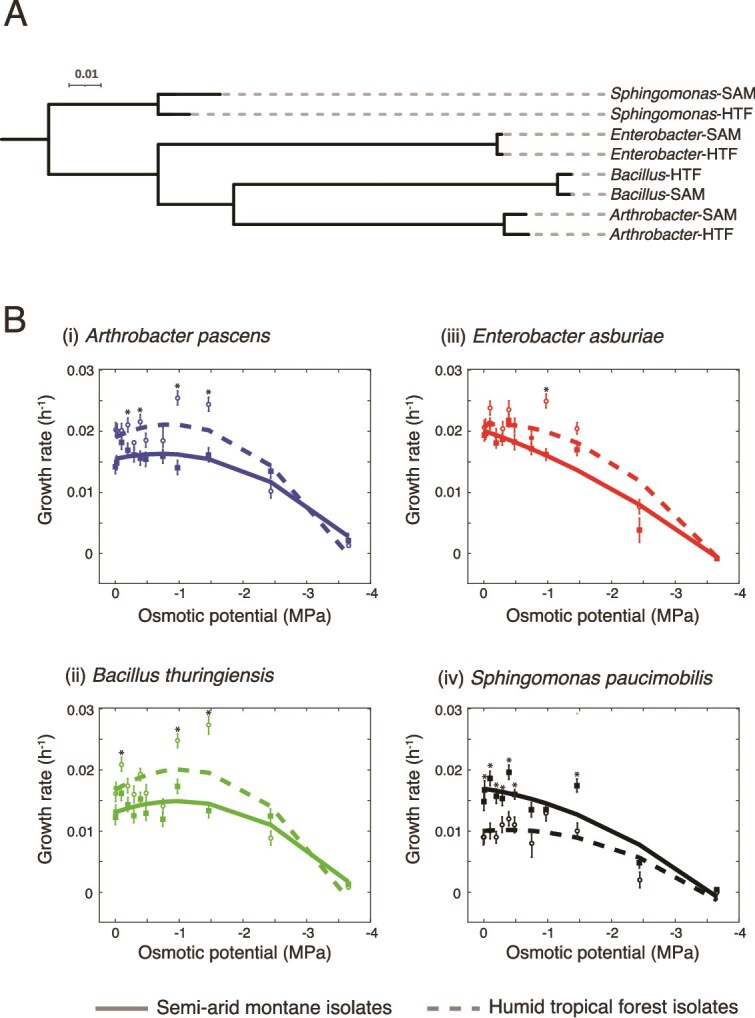
Phylogeny and growth of the different isolates. (A) Phylogenetic relationships between the various isolates (the scale is indicative of phylogenetic changes). (B) Growth rates across a range of solute potential measurements for (i) *Arthrobacter pascens*, (ii) *bacillus thuringiensis*, (iii) *Enterobacter asburiae*, and (iv) *Sphingomonas paucimobilis*. The rates are shown as a second-order polynomial that best fits the data. The asterisks represent statistically significant differences (*P* < 0.05) in growth rates between the two isolates.

To explore whether broad differences in stress response were apparent between bacteria isolated from the different soils, we first measured survival of cells undergoing the MS and OS experiments using flow cytometry. Under these conditions no significant differences were noted in survivorship between the control and experimental treatments, with >97% of cells alive following exposure to either of the stress conditions (Supplementary Fig. 5), demonstrating the non-lethal nature of the current experiments. Next, we measured the growth rate of the different isolates under increasing concentrations of NaCl (between 2 and 750 mM, spanning an added solute potential gradient of ~0 to −3.6 MPa). The HTF isolates, with the exception of the *Sphingomonas*-HTF, showed significantly higher growth rates than the SAM isolates, but greater sensitivity to increasing solute potential (Fig. 2B). In general, increasing solute concentration imparted a threshold effect on growth rate, whereby osmotic potential greater than −1 MPa resulted in declining growth rates (Fig. 2B). However, the Gram-positive isolates from the SAM soils had the highest growth rates at highest solute concentrations, and a gentler decline in growth rate overall.

### Diversity of biochemical phenotype under osmotic and matric stress

**Figure 3:**
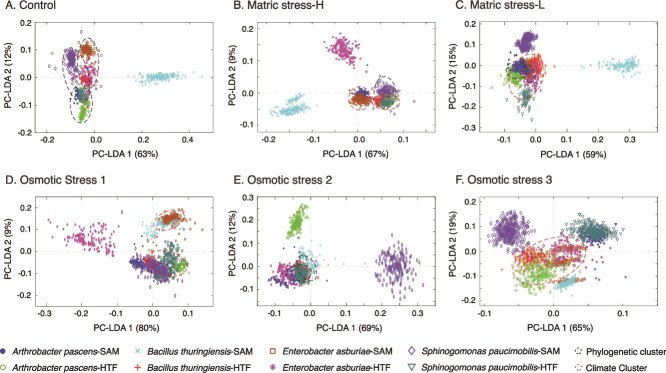
Ordination plots based on principal component-linear discrimination analysis derived from infrared spectral biochemical fingerprints of multiple cells within (A) the control experiments, and (B–F) the different stress experiments. Each point on the ordination represents cells within a 5–10 micron diameter spot area, on average 20 cells are scanned within each spot, given a total of between 300 to 400 cells scanned for each isolate per experiment. Isolates clustering either by phylogeny or climate are highlighted on the respective panels.

We report briefly on how the broader biochemical phenotype (*i.e.* the collective expression of metabolism) manifests under control and stress conditions (Fig. 3). A detailed account of the biochemical factors separating different isolates under these conditions is provided in the supplementary material. Bacterial microcolonies under control conditions generally cluster by phylogeny, with separation by cell wall structure (i.e. Gram-positive or -negative) and intracellular energy storage polymers (Fig. 3A, Supplementary Fig. 6a). However, this pattern of separation based on cell-wall structure and the energy storage polymers does not always hold under the different stress treatments (Fig. 3B–F). There was heterogeneity in the stress response unrelated to cell-wall structure. Under several conditions, including MS-H, OS1, and OS3, organisms of different phylogeny isolated from sites with the same climate history (i.e. SAM or HTF) show a more similar response to stress, clustering together on the ordination. Conversely, there is also evidence for a similar response based on phylogeny rather than climate history for the MS-L and OS2 experiments.

### Changes to the biochemical phenotype under stress

**Figure 4:**
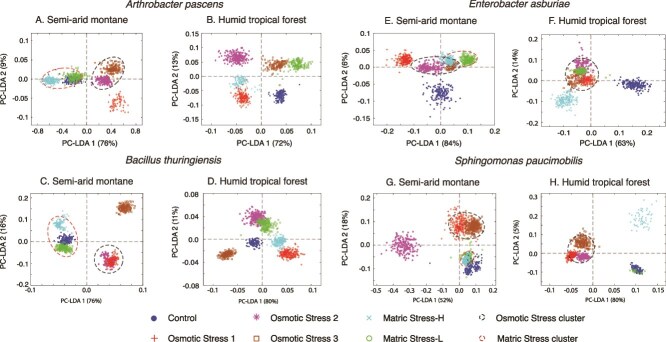
Ordination plots based on principal component-linear discrimination analysis for each isolate. Each panel shows the ordination of the biochemical phenotype under control and different experimental conditions of osmotic and matric stress. The panels provide results for bacteria isolated from SAM and HTF soils for the gram-positive *A. pascens* (A/B), and *bacillus thuringiensis* (C/D), and the gram-negative *E. asburiae* (E/F), and *S. paucimobilis* (G/H). The dashed ovals cluster similar experimental responses to either osmotic stress or matric stress.

The majority of variance (>80%) in the biochemical phenotype of the cells undergoing OS or MS stress (and the corresponding controls) is captured by the first two factors of the PC-LDA analysis (with the first 3 PC-LDA components explaining over >90%, Supplementary Fig. 7), allowing us to focus on inter-treatment variation for all eight isolates (Fig. 4).

The short-pulsed nature of the stress experiments was capable of provoking a metabolic response in each of the isolates, however, the divergence in biochemical phenotype between the treatments and control was dependent on the climate history of an isolate (Fig. 4). For example, the biochemical phenotype of experimentally stressed isolates from tropical forest soils never overlapped with the control phenotype (Fig. 4B, D, F, H), even under the mild matric stress experiments. This is demonstrated by Gram-positive *Arthrobacter*-HTF and *Bacillus*-HTF, and the Gram-negative *Enterobacter*-HTF, where a unique biochemical phenotype was observed for the control and each of the stress conditions (Fig. 4B). For *Arthrobacter*-HTF the first two PC-LDA components separated the matric stress (MS-H, MS-L) from osmotic stress experiments (OS1, OS2, OS3), as well as high stress (MS-H, OS2, OS3) from low stress experiments (MS-L, OS1). *Bacillus*-HTF showed clustering of OS and MS experiments (e.g. MS-H and OS1), with OS2 diverging along PC-LDA1. The biochemical phenotype of *Enterobacter*-HTF clustered for the OS experiments, with MS-H ordinating away from the other experiments along PC-LDA1. For *Sphingomonas*-HTF (Fig. 4H), the matric and osmotic stresses were separated along the primary PC-LDA, with a strong cluster of OS experiments; however, there was overlap between the control experiments and MS-L.

Unlike the HTF isolates, those from semi-arid soils showed strong overlap between the biochemical phenotypes of matric stress experiments and the controls (Fig. 4A, C, E, G). The *Arthrobacter* and *Bacillus*-SAM isolates showed co-clustering of the matric stress and control biochemistry, and separation from the osmotic stresses along the primary axis PC-LDA1 (Fig. 4A, C), which accounted for a majority of variance (76%). For the Gram-negative *Sphingomonas*-SAM, matric stress and control biochemistry co-clustered and were separated from osmotic stresses along the secondary PC-LDA axis (Fig. 4G). Finally, the *Enterobacter*-SAM control phenotype exhibited minimal overlap between the various stress experiments (Fig. 4E), similar to that of the *Enterobacter*-HTF isolate.

### Quantitative analysis of isolate-specific response to different stress

To highlight the broad responses of different isolates to stress, we analyzed the lipid/ fatty acid region (~1760–1600 cm^−1^), the phosphorus bond region (1230–1200 cm^−1^), and the carbohydrate region (1250–1000 cm^−1^, Supplemental Figs. 8 and 9). The phosphorus bond region can be indicative of the presence or absence of energy storage polymers, and molecules involved in two-component signal transduction systems, which consist of >PO_2_^−^ groups. We also contextualize these broad responses with data on specific storage compounds (e.g. PHAs, PHBs, polyphosphates, and glycogen), stress related metabolites (e.g. trehalose, glutamate and proline) and osmolytes (e.g. mannitol, ectoine, and glycine betaine). Detailed analysis of band position shifts relative to the control spectra is provided in the Supplementary Tables 4 to 11.

**Figure 5:**
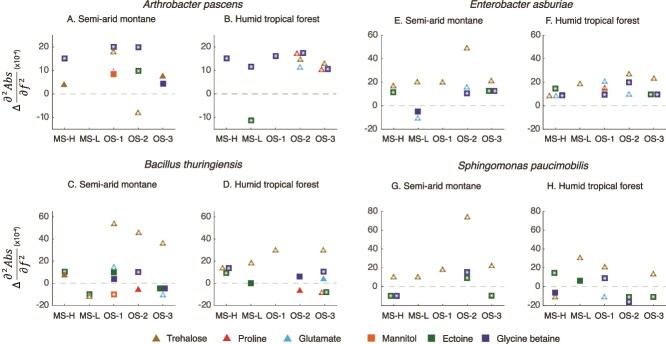
Changes in the relative concentration of different bacterial stress metabolites and osmolytes under matric and osmotic stress for each isolate. The general stress metabolites (denoted by triangles) include trehalose, proline, and glutamate, whereas those associated with osmotic stress are represented by square points, and include mannitol, ectoine, and glycine betaine. These compounds have previously been shown to increase under drought conditions. The y-axis is of the same magnitude for each of the isolate pairs, but differs between isolates. White asterisks denote those metabolites that differ from the control to a statistically significant extent (*P* < 0.05). Standard deviation is represented by a 2σ error, however the standard error bars are often masked by the point size.

**Figure 6:**
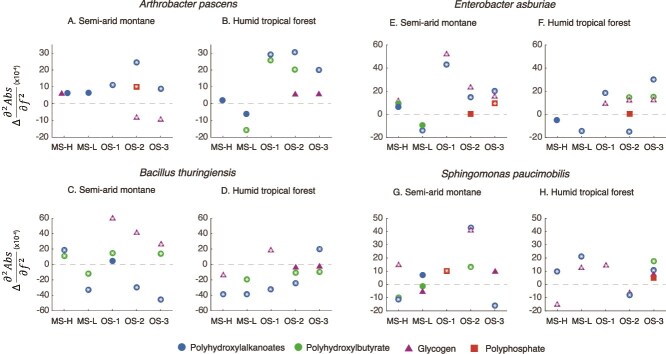
Specific metabolic shifts in energy storage molecules (polyhydroxyalkanoates, polyhydroxybutyrates, glycogen, and polyphosphate) under matric and osmotic stress. The y-axis is of the same magnitude for each of the isolate pairs but differs between isolates. White asterisks denote those metabolites that differ from the control to a statistically significant extent (*P* < 0.05). Standard deviation is represented by a 2σ error; however, the standard error bars are often masked by the point size.

The biochemical response of the SAM isolates to MS-H stress showed an overall increase in the carbohydrate (1200–1000 cm^−1^) compared to the controls (Supplemental Fig. 8). This increase is linked to higher osmolyte production (Fig. 5) and an accumulation of carbohydrate storage compounds like glycogen (Fig. 6). Although the lipid region showed variable response, there was a general increase in the PHAs under stress (Fig. 6). In contrast, HTF isolates showed an overall upregulation of the phosphate (υPO_2_^−^) region (1230–1200 cm^−1^), and a general decline in the lipid and carbohydrate regions (Supplemental Fig. 8). This broader decline in lipids was accompanied by a strong decrease in PHA storage compounds. Glycogen also decreased for *Bacillus* and *Sphingomonas*, however, there was a general increase in some osmolytes, mainly glycine betaine, but also trehalose and ectoine (Fig. 5).

Under the mild MS experiment, MS-L, isolates from most semi-arid soils showed consistent overlap between the emergent biochemical response under matric stress and the control biochemical phenotype (Fig. 4A, C, G). This emphasizes that MS-L did not impart significant stress on these isolates. Although there was a broad increase in carbohydrate production (Supplemental Fig. 8), the osmolyte response was generally weak, with only some evidence of trehalose production in *Enterobacter* and *Sphingomonas* isolates, and no production of osmolytes in *Arthrobacter*-SAM (Fig. 5). In contrast, isolates from humid tropical forest soils displayed a more divergent response under MS-L compared to control cells, characterized by increased production of carbohydrates and a decline in lipids (Supplemental Fig. 8). Specifically, PHA storage declined in most isolates as did most osmolytes (Fig. 6). The exception here was *Sphingomonas*-HTF, which increased the production of PHA relative to the control, and increased concentrations of glycogen, trehalose, and ectoine.

Several isolates, particularly from semi-arid soils (e.g. Arthobacter-SAM, *Bacillus*-SAM, and *Sphingomonas*-SAM) as well as *Sphingomonas*-HTF, showed distinct biochemical responses to osmotic stress compared to matric stress. Osmotic stress experiments produced unique biochemical responses across the SAM isolates relative to controls (Fig. 4A,C,G,H), with a broad decrease in the lipid region, and increase in the carbohydrate region. Under osmotic stress 1 (40 mM), the decrease in the lipid region cannot be explained by changes in storage compounds, as we observed a general stress-induced increase in PHA, PHB, and polyphosphate across all isolates (Fig. 6). The broader increase in carbohydrate was mirrored by a general rise in osmolytes of multiple types (Fig. 5). In contrast, the majority of HTF isolates (*Arthrobacter*-HTF, *Bacillus*-HTF, and *Sphingomonas*-HTF), showed a broad decrease in lipid and carbohydrate regions, with a concomitant increase in the υPO_2_^−^ region (Supplemental Fig. 9). Despite the broader drop in carbohydrate, there was a strong increase in stress-related osmolytes, mainly trehalose, and glycine betaine.

Preconditioning the cells to lower osmotic stress before subjecting them to higher stress (OS2) prompted two major responses, (i) an intermediate biochemical response clustering between the OS1 and OS3 phenotypes (Fig. 4A, E, H), and (ii) several unique responses in which the OS2 experiment clusters away from the other OS experiments (Fig. 4B, D, F, G). For the SAM isolates OS2 resulted in a decline or no change in the carbohydrate regions, distinct from the OS1 response. (Supplemental Fig. 9). The lipid region response varied among isolates. In general, there was a similarly strong osmolyte response under OS2 as that observed in OS1, with glycine betaine a particularly prominent compound increasing under stress. Similarly, the storage compounds generally increased under the OS2 treatment, with PHA increasing within most isolates. In instances where PHA declined (e.g. *Bacillus*-SAM) there was a concomitant increase in glycogen.

For the HTF isolates, including *Arthrobacter*-HTF, *Enterobacter*-HTF and *Sphingomonas*-HTF, we observed a similar decrease in the carbohydrate region during OS2 as seen in the OS1 experiment (Supplemental Fig. 9). This was accompanied by an increase in osmolyte production, mainly glycine betaine (Fig. 5). In contrast, *Bacillus*-HTF showed an increase in the carbohydrate region, particularly in glycine betaine, under the step-up experiment. For most HTF isolates, this strong increase in osmolytes was accompanied by a decline in cellular storage compounds, including glycogen and PHA.

Doubling the NaCl concentration to 80 mM (OS3) provoked a strong elevation in the carbohydrate region for the HTF isolates (Supplemental Fig. 9), a decline in the broader lipid region, and a generally negligible response in the υPO_2_^−^ region for most isolates. When considering distinct spectral features, most of the SAM and HTF isolates respond to the OS3 treatment through the accumulation of PHA (and in some cases PHB and polyphosphate), and the production of osmolytes. Trehalose increased in every isolate compared to control conditions (Fig. 5).

## Discussion

Soil microorganisms inhabit dynamic environments where rapid fluctuations in soil moisture are common [2]. During periods of hypo- and hyperosmolarity, prokaryotes must maintain positive intracellular turgor pressure to sustain growth and cell division [33]. The SR-FTIR spectromicroscopy approach confirms the key role of osmolyte production under osmotic stress and highlights the importance of storage polymers and cell–cell signaling (υPO_2_^−^ region) as responses to stress. We synthesize the data below to address the overarching hypotheses of this study.

### Onset of matric and osmotic stress elicit distinct metabolic responses in bacteria

Our initial hypothesis proposed that the different constituents of drought—matric stress and osmotic stress—will impart distinct metabolic responses in bacteria. Support for this hypothesis comes predominantly from the SAM isolates, which show that distinct biochemical phenotypes emerge from the matric and osmotic stress experiments (Fig. 4). An extensive analysis of the biochemical phenotype shows stress metabolites were more prominent under the OS experiments, although little response to the MS experiments was observed: *Arthrobacter*-SAM and *Bacillus*-SAM showed no clear deviation from the control phenotype under MS-L, whereas *Sphingomonas* and *Enterobacter*-SAM exhibited only moderate responses compared to the response to the OS treatments. These observations emphasize that organisms isolated from semi-arid soils are well acclimated to acute osmotic stress, reflecting their evolutionary adaptations to seasonal drought and to freeze–thaw cycles, which increase the osmotic potential of interstitial pore space [34]. In contrast, HTF isolates exhibited more similarity between the MS and OS experiments, and the responses rarely overlapping with control conditions. This suggests a more restricted range of stress phenotypes within these isolates.

Our analysis also highlights similar responses to stress for the isolates. The upregulation of cell signaling (represented by the υPO_2_^−^ region) was observed in most isolates under matric and osmotic stress experiments. These pathways commonly regulate bacterial response in dynamic environments [35], preceding the general stress response of bacteria [36], including the upregulation of the secondary metabolome [37] and biofilm production [38], all of which are important mechanisms mitigating the impact of osmotic stress on bacteria.

The SR-FTIR data also demonstrated increases and decreases in lipid storage polymers (i.e. PHAs and PHBs) and glycogen, a carbohydrate storage compound, under matric and osmotic stress. These compounds, which were particularly conspicuous under OS conditions (Supplemental Fig. 10), are widely distributed amongst bacteria [39], including within taxa from the present study [40–42]. The production and accumulation of storage polymers enable cells to survive periods of stress [43]. Starvation is a primary trigger upregulating the re-allocation of resources to the production of storage compounds [39], and changes in osmotic potential under drought are often accompanied by a drop in substrate diffusion through soil pores [1], resulting in cell starvation [2]. However, because our experiments were conducted under carbon replete conditions, the accumulation of storage compounds may result from a conditioned response to cells experiencing fluctuating osmotic potential. Indeed, storage polymers are themselves important metabolic responses for protecting cellular integrity under osmotic stress [44, 45].

The observed production of polysaccharides, amino acids and osmolytes under stress was a common response within both semi-arid and tropical forest isolates exposed to osmotic stress, and soils undergoing drought, consistent with previous work [15, 46, 47]. The OS treatments tended to provoke a higher diversity of metabolites relative to the MS treatments, with multiple metabolites (e.g. trehalose, ectoine, proline, and glycine betaine) produced under each of the OS treatments (Fig. 5), suggesting that more complex pathways of response are necessary under more stressful conditions.

The prominent role trehalose plays under the MS and OS conditions might be attributed to it being a general stress metabolite (Fig. 5). Trehalose production protects protein structure and membrane integrity and is commonly upregulated under various stress conditions [5, 48, 49]. However, trehalose plays a multifaceted role in stress, also functioning as a storage compound [39], and serves as an important substrate post-stress [50]. Given that compatible solute production is an energetically expensive process [51], requiring the re-allocation or novel acquisition of resources to support synthesis, the upregulation of a versatile compound serves several requirements in a cells response to stress. Glycine betaine and ectoine also played an important role in the overall stress response. In this case, both likely function as osmoprotectants, and are upregulated in response to changing osmotic potential [52]. For both *Arthrobacter* isolates glycine betaine was the primary metabolic response to stress, which suggests a stress response pathway conserved on evolutionary timescales.

### Climate history will shape the metabolic response to stress

Our second hypothesis addressed the role climate history plays in determining the microbial response to stress. Climate history plays a critical role in shaping the adaptive capacity of microorganisms [17, 53], and we hypothesized here that isolates from semi-arid soils adapted to long seasonal drought would respond through a stronger metabolic response than isolates from humid tropical forest soils. The evidence supporting or refuting this hypothesis is complex. SAM isolates grow at a higher rate than HTF isolates under lower osmotic potentials (Fig. 2b) and maintained a biochemical phenotype similar to the control under matric stress (Fig. 4). They also showed a pronounced response to OS, with higher abundances of stress metabolites such as trehalose and ectoine when compared to the HTF isolates (Fig. 5). Indeed, the climate-specific production of storage and stress compounds were consistently higher than control conditions for the SAM isolates, but not for the HTF isolates (Supplementary Fig. 8). Thus, this study highlights that SAM isolates, shaped by long-term drought legacies, show strong and more pronounced metabolic responses to stress than HTF, consistent with eco-evolutionary adaptation of microorganisms [54].

We investigated how pre-exposure to moderate stress would change the biochemical phenotype to more severe stress. Previous studies have shown bacteria to anticipate changes in their local environment, and respond accordingly [6, 19]. In the current study, we exposed cells to lower concentrations of NaCl prior to doubling the concentration. The overlap between OS2 and the other OS experiments (Fig. 4A, C, E, F, H) demonstrates a conserved response to osmotic stress, consistent with a general stress response [36]. However, in several cases the observed biochemical phenotype showed a response distinctive from that of either OS1 or OS3 (Fig. 4B, D, G). For *Arthrobacter*-HTF and *Sphingomonas*-SAM a stronger and more diverse metabolic response was noted for OS2 in both the stress metabolites (Fig. 5), and the storage compounds (Fig. 6), relative to OS1 and 3.

## Conclusions

The current study characterizes the biochemical phenotype of bacteria isolated from semi-arid and tropical forest soils under matric and osmotic stress. We observed the response to stress is dependent on the nature of the stress, but also the climate history of the individual isolate, with SAM isolates showing greater adaptation to fluctuating environmental conditions. However, we recognize limitations with the current study. The findings are limited to a small number of isolates that are represented at both sites. These findings are not, therefore, representative of the microbial community as a whole, which can show extremely divergent responses to soil moisture stress both within and between phyla [55].

We believe that the SR-FTIR technique can be developed further to become a valuable approach for non-destructively profiling microbial physiological responses to stress. Recent developments in the field of microfluidics permits coupling to SR-FTIR through flexibility in material usage [56, 57], whereby, polydimethylsiloxane constructed devices, ordinarily incompatible with the synchrotron light source, includes an includes an infrared-transparent or virtual window permitting sustainable detection of FTIR spectral signals [57, 58]. Microfluidics offer precise regulation of the chemical and physical environments and gradient formation [59], allowing for the experimental simulation of compound disturbances (e.g. osmotic stress during warming). Combing microfluidic technologies with SR-FTIR offers a detailed mechanistic basis to observations of spatial shifts, and these experiments enable an improved understanding of cell–cell interactions, or deterministic interactions, which increase under soil drying [60], and are likely as significant, if not more significant, in adaptation, co-existence, regulating the biochemical phenotype under stress [61, 62]. Such interactions should be the focus of future research to determine how communities respond to a changing climate.

## Supplementary Material

Supplemental_Climate_history_FTIR_wraf075

## Data Availability

In accordance with US-DOE data policy, the data presented in this manuscript is available in the ESS-DIVE repository (https://ess-dive/gov) under doi:10.15485/2549408.
