# Supplementary material for: Climate history modulates stress responses of common soil bacteria under experimental drought
Source: ISME J. 2025 Apr 18;19(1):wraf075. doi: 10.1093/ismejo/wraf075 (PMC12085270; doi:10.1093/ismejo/wraf075)
Supplement: Supplemental_Climate_history_FTIR_wraf075 [file supplemental_climate_history_ftir_wraf075.pdf]

Supplementary Material

Climate History Modulates Stress Responses of Common Soil Bacteria Under Experimental Drought

Nicholas J. Bouskill[1,2*], Stephany S. Chacon[1,3], Daniela F. Cusack[4,5], Lee H. Dietterich[4,6], Liang Chen[7], Aizah Khurram[1], Jana Voriskova[1,8], Hoi-Ying N. Holman[7,9]

[1]Climate and Ecosystem Sciences Division, Lawrence Berkeley National Laboratory, Berkeley, CA, 94720. *Corresponding author: njbouskill@lbl.gov

[2]Department of Biological and Ecological Engineering, Oregon State University, Corvallis, OR, 97330, USA.

[3]Life and Environmental Science Department, University of California Merced, Merced, CA, 95343, USA.

[4]Department of Ecosystem Science and Sustainability, Colorado State University, Campus Delivery 1476, Fort Collins, Colorado, 80523.

[5]Smithsonian Tropical Research Institute, Apartado, 0843-03092 Balboa, Ancon, Republic of Panama.

[6]Department of Biology, Haverford College, PA, USA.

[7]Berkeley Synchrotron Infrared Structural Biology Program, Lawrence Berkeley National Laboratory, Berkeley, CA, 94720.

[8]Institute of Microbiology, Czech Academy of Sciences, Vídeňská 1083, 14220 Praha 4, Czech Republic.

[9]Molecular Biophysics and Integrated Bioimaging Division, Lawrence Berkeley National Laboratory, Berkeley, CA, 94720.

Supplementary Material

Supplemental materials & methods……………………………………………………….3

Supplemental results……………………………………………………………………5

Supplemental figures…………………………………………………………..…10

Supplemental tables……………………………………………………………………23

References……………………………………………………………………………...67

Supplemental Material & Methods

Isolations: Isolation of the soil bacteria proceeded according to previously published approaches [1]. Briefly, 10 g of soil was re-suspended and serially diluted ten-fold in a sterile saline solution (0.95 % NaCl). 0.1 ml of each dilution was plated onto one of five solid media, including various dilutions of R2A, R2G, DNBG, ISS, and the oligotrophic media, M9. The specific recipes for each media are provided in the Supplementary Table 1.

The solid media was incubated for eight weeks, and colonies picked as they grew. To construct a diverse isolate library, we took an indiscriminate approach to colony picking, before serially diluting the colony in liquid media to ensure a pure culture. The lowest dilutions were re-plated on the initial media and regrown for 48 hours. Isolates were re-suspended in liquid media and grown overnight. A 0.5 ml aliquot of the axenic culture was vortexed with 0.5 ml glycerol and stored at -80 °C. Glycerol stocks were all regrown within an M9 liquid media (0.16 M Na_2HPO_4, 0.11 M KH_2PO_4, 0.095 M NH_4Cl, 0.043 M NaCl, $2x10^{-3}$ M $MgSO_4$, $2x10^{-4}$ M $CaCl_2$, 20 % glucose), to standardize media conditions for synchrotron experiments.

Nucleic acid extraction and 16S rRNA sequencing: DNA was extracted from the axenic cultures using the *Quick*-DNA Fungal/ Bacterial Miniprep kit (Zymo Research, CA, USA) according to the manufacturer's protocol. 10 ng of genomic DNA was amplified in 25 µl PCR reactions containing 400 nM of each primer, 27F [2] and 1492R [3]; and 2.5 U of DreamTaq Master Mix (Thermofisher Scientific, MA, USA). To obtain SSU rRNA gene sequences, PCR products were sequenced from both ends using primers 27F [2] and 1492R [3] at the UC Berkeley DNA Sequencing Facility (Berkeley, CA, USA). Templates were amplified using standard PCR conditions (95 °C x 5 mins, 30 cycles: 95°C, 52°C, 72°C each for 30 seconds, 72°C for 10 minutes).

A consensus sequence for each read was assembled (Geneious v10.11), and compared to the BLAST database to assign their taxonomic identity. The corresponding FASTA sequences were then aligned using a multiple sequence alignment program (MAFFT 7.0) and visualized in ITOL [4]. Further details of the isolates used in the present study are provided in Supplementary Table 1. For ease of reference, the isolates were named from both their broad genus name and the site

of isolation (either SAM or HTF). For example, *Arthobacter Pascens* from the semi-arid montane site would become Arthobacter-SAM.

Supplemental Results

Diversity in biochemical phenotypes under stress-free control conditions: Bacterial microcolonies without induced stress generally cluster by phylogeny, with separation by cell wall structure and intracellular energy storage polymers (Fig. 3a, Supplementary Fig. 6a). The Gram-positive Bacillus from semi-arid soils (Bacillus-SAM) exhibits distinct biochemistry relative to all other isolates along the primary axis (PC-LDA1), which accounted for ~64 % of the variance. This axis is primarily defined by spectral features affiliated with bacterial energy storage polymer mixtures, such as polyhydroxyalkanotes (PHA), polyhydroxybutyrates (PHB), and energy yielding phospholipids (Supplementary Fig. 11f). The largest contribution to the separation lies in the lipid/fatty acid hydrogen-bonded >C=O region 1750-1710 cm^{-1}, which consisted of several overlapping peaks (Supplementary Fig. 12a) at ~1745 cm^{-1} , ~1740 cm^{-1}, and 1732 cm^{-1} assigned to υ(>C=O) of the ester within PHB or within PHA and PHB mixtures[26,27]. The largest bands at ~1730 cm^{-1} were assigned to υ(>C=O) of the carboxylic acid (–COOH) of phospholipids with additional supporting contributions from protein amide II (~1542 cm^{-1}, 1518 cm^{-1}), δ_{as} and δ_{s} (C–H) of CH_3 and CH_2 (~1453 cm^{-1}, ~1338 cm^{-1}), υC-O of ester (~1290 cm^{-1}), υ_{as} (>PO_2^-) at ~1240-1220 cm^{-1} and υ_{s} (>PO_2^-) at ~1090 - 1080 cm^{-1} of phospholipids and nucleic acids, and overlapping υ (C-O-C) and υ(P-O-C) at 1170 - 1160 cm^{-1} of carbohydrates[28–30].

The secondary axis (PC-LDA2), which accounted for ~13% of the total variance, was largely defined by absorbance of molecular features associated with cell wall structure (Supplementary Fig. 6a). Comparison of the secondary derivative spectra (Supplementary Fig. 2a) with the PC-LDA2 loading spectra (Supplementary Fig. 6a) show a number of spectral bands from the Gram-positive bacteria that can be assigned to the functional groups of bacterial cell-wall component peptidoglycan. The bands at ~1725-1705 cm^{-1} are assigned to hydrogen-bonded carbonyl groups υ (>C=O) of aldehydes, ketone and carboxylic acids, while those at ~1600 cm^{-1} and ~1400 cm^{-1} relate to υ_{as} and υ_{s} (>C=O) of carboxylate (– COO^-) end or side chains of peptidoglycan. The bands at ~1659 cm^{-1} and ~1542 cm^{-1} are amide I and amide II of proteins arising in part from amino acids, whereas bands at ~1455 cm^{-1} and ~1380 cm^{-1} are δ_{as} and δ_{s} (C–H) of CH_3 and CH_2, respectively. The bands at ~1223 cm^{-1} and ~1075 cm^{-1} are υ_{as} and υ_{s} (>PO_2^-) of phospholipids

and nucleic acids. Finally, bands of the complex sugar aromatic ring vibration modes are at ~1150, ~1060 cm^{-1}, and 1045 cm^{-1} [29,31,32].

Multivariate discriminative analysis showed bacteria from the same phylogeny but different climate ecosystems share similar biochemical composition. The cluster vector spectra for the two Arthrobacter isolates, for example, showed overlapping biochemical phenotypes under stress-free conditions, as shown by the contemporaneous peaks in the 1800-1000 cm^{-1} region, albeit at different peak intensities (Supplementary Fig. 3a). This was also the case for the two Sphinogomonas and Enterobacter isolates. The greatest distance between paired isolates was observed for the Bacillus, with infrared absorbance from Bacillus-SAM showing a distinct abundance in energy storage polymers relative to Bacillus-HTF.

Under the high matric stress treatment (MS-H) the score plots showed a cluster (Fig. 3b) which exhibits a large degree of overlap among three tropical isolates the Gram-positive Bacillus-HTF, Arthrobacter-HTF, and the Gram-negative Sphingomonas-HTF. The accompanying vector plots (Supplementary Fig. 3b) show small differences among these three tropical isolates, lower absorbance in the 1770 - 1720 cm^{-1} spectral region of lipids, 1570-1510 cm^{-1} of amide II, 1490 - CH_2 and CH_3 and higher absorbance in the υPO_2^- (~1250-1200 cm^{-1}) region. These overlapping tropical isolates are complimented by three adjacent semi-arid isolates, the Gram-negative Enterobacter-SAM, Sphingomonas-SAM, and the Gram-positive Arthrobacter-SAM. The cluster vectors of Enterobacter-SAM and Arthrobacter-SAM show lower absorbance at ~1730 cm^{-1} in the lipid region and much higher absorbance at ~1110 cm^{-1} and ~1076 cm^{-1} in the carbohydrate region. In contrast, the cluster vector of Sphingomonas-SAM shows lower absorbance at ~1730 cm^{-1} but much higher absorbance at 1214 cm^{-1}, similar to Sphingomonas-HTF. The final two isolates, Bacillus-SAM, which split into two subgroups, and Enterobacter-HTF, ordinate away from the main cluster and each other. The most dominant wavenumbers in distinguishing Bacillus-SAM and Enterobacter-HTF from the other isolates are the large absorbance at ~1730 cm^{-1} and ~1155 cm^{-1} respectively. Moreover, the vector spectra show that the discrepancy between the two Bacillus isolates from different climate ecosystems lies in divergent responses in the lipid/fatty acid, proteins, and carbohydrate regions, with prominent increases at 1730, 1664, 1490-1390, 1295, 1135, 1050-1010 cm^{-1} for Bacillus-SAM, and decreases at 1710, 1598,

1215, and 1076 cm^{-1}. Most of the difference between Enterobacter-HTF and Enterobacter-SAM can be explained by differences in the carbohydrate (1250-1100 cm^{-1}) region, with important contributions from amide I /II.

Even under the milder MS experiment (MS-L), we observed a similar cluster of tropical isolates (Arthrobacter-HTF, Bacillus-HTF, and Enterobacter-HTF), alongside the phylogenetically similar semi-arid counterparts for Arthobacter-SAM and Enterobacter-SAM (Fig. 3c). The cluster vector plots (Supplementary 3c) show small differences among these tropical isolates, or the semi-arid isolates except for the higher absorbance at ~1648 cm^{-1}, ~1496 cm^{-1}, ~1400 cm^{-1}, and ~1223 cm^{-1} in the Arthrobacter-SAM isolate. The Sphingomonas isolates ordinate away from this large cluster, and directly opposite one another. This implies unique spectral responses to treatment between these two isolates, with significant contributions from Sphingomonas-HTF and Sphingomonas-SAM at ~ 1102 cm^{-1} and ~1020 cm^{-1} (carboxylates) respectively. The Bacillus-SAM ordinated away from the Bacillus-HTF (and all isolates), predominantly due to major contributions from the high lipid peak at ~1740 cm^{-1} and the double carbohydrate peaks at ~1136 cm^{-1} and ~1028 cm^{-1}, and the low absorbance at ~1630 cm^{-1} (protein amide I), ~1225 cm^{-1}, and ~1075 cm^{-1}.

The clustering emerging under osmotic stress experiments was related to both phylogeny and climate history (Fig. 3d, e, f). Climate history tended to guide the biochemical response under direct exposure (i.e., OS1 & OS3), while the step-up experiment (OS2) with priming "osmotic stress" resulted in a response clustering by phylogeny. A strong cluster of multiple isolates emerged under the milder OS experiment (OS1). Furthermore, the most severe OS experiment (OS3) also showed evidence for the emergence of unique biochemical phenotypes for each isolate, with the HTF cluster more diffuse under this condition (Fig. 3f).

We found that bacterial exposure to osmotic stress resulted in fundamentally different changes (relative to matric stress and the stress-free controls) in cell biochemistry. Under OS1, the score plots (Fig 3d) and cluster vector spectra (Supplementary Fig. 3d) illustrate a large degree of co-clustering and overlap. The tropical isolates Arthobacter-HTF, Bacillus-HTF, and Sphingomonas-HTF showed biochemical response similarity (Fig. 3d), with showing higher

amplitude peaks at ~1724 cm^{-1}, ~1710 cm^{-1}, ~1593 cm^{-1}, ~1216 cm^{-1}, ~1186 cm^{-1}, ~1063 cm^{-1}, and ~1056 cm^{-1} in Arthrobacter-HTF. The semi-arid isolates, Bacillus-SAM and Enterobacter-SAM showed overlapping peaks with little differences. Similarly, Arthrobacter-SAM and Sphingomonas-SAM co-clustered, however, the amplitude peaks of Arthrobacter-SAM at ~1730 cm^{-1}, ~1710 cm^{-1}, ~1690 cm^{-1}, ~1498 cm^{-1}, ~1225 cm^{-1} and ~1125 cm^{-1} were larger. Finally, the tropical Enterobacter-HTF ordinated alone along the primary axis which accounted for most (80%) of the variations, and the secondary axis which accounted for 9% of the variations. This distinct separation was defined by a significant absorption peak at ~1110 cm^{-1} (Supplementary Fig. 3d).

A subsequent priming OS experiment (OS2), that pre-conditions the isolates to the lower osmotic stress (i.e., OS1) before exposure to higher stress, revealed the emergence of a co-cluster of bacterial isolates showing similar biochemical responses to the step-wise experiment (Fig. 3e). This large co-cluster again showed similarity in the responses of phylogenetically similar organisms isolated from different environments, containing both the tropical isolates Bacillus-HTF Sphingomonas-HTF and semi-arid isolates Bacillus-SAM, Enterobacter-SAM, and Arthrobacter-SAM, the latter was split into two small subgroups. The cluster vectors of Bacillus-HTF, Enterobacter-HTF, and Arthrobacter-SAM in Supplementary Fig. 3c showed overlapping peaks at ~1466 cm^{-1}, ~1444 cm^{-1}, ~1230 cm^{-1}, ~1221 cm^{-1}, and ~1120 cm^{-1}. Enterobacter-HTF and Arthrobacter-SAM showed overlapping peaks at ~1523 cm^{-1} (amide II), ~1225 cm^{-1} (υPO_2^-), ~1100 cm^{-1}, and ~1068cm^{-1} (carbohydrates). The important feature in distinguishing Arthrobacter-HTF from this 6-member cluster along the secondary axis (PC-LDA2) was the large amplitudes of peaks at ~1466 cm^{-1}, ~1444 cm^{-1}, ~1230 cm^{-1}, ~1221 cm^{-1}, and ~1120 cm^{-1}. Sphingomonas-SAM differed from all others across the primary axis (PC-LDA1), which accounted for 69% of the variations. The vector plot showed a large difference between Sphingomonas-SAM and all other isolates within the lipid region (~1745 cm^{-1}), amide I (~1662 cm^{-1}, ~1630 cm^{-1}, ~1625 cm^{-1}), δ_{as} and δ_s (C–H) of CH_3 and CH_2 in lipids and proteins (~1488 cm^{-1}, ~1440 cm^{-1}, ~1364 cm^{-1}, ~1337 cm^{-1}, ~1326 cm^{-1}, ~1316 cm^{-1}, and carbohydrates (~1180 cm^{-1}, ~1163 cm^{-1}, ~1154 cm^{-1}, ~1066 cm^{-1}, ~1053 cm^{-1}).

Finally, relatively unique biochemical responses emerged under the most severe OS experiment (OS3). The Enterobacter isolates show some overlap in their response, but this might be attributed to a heterogeneous response of different microcolonies of Enterobacter-HTF, which shows clear variability rather than a succinct cluster. In general, the response to OS3 produces strong heterogeneity across the isolates as they respond to rapid changes in external osmotic pressure (Fig. 3f). Here the semi-arid Gram-negative Sphingomonas-SAM and the two tropical Gram-positive isolates Arthrobacter-HTF and Bacillus-HTF differed from the others along the primary PC-LDA axis that accounted for 65% of the variation in the OS3 data, which was largely defined by the absorbance in amide I and II (~1694 cm^{-1}, ~1653 cm^{-1}, ~1635 cm^{-1}, 1548 cm^{-1}), δ_{as} and δ_{s} (C–H) of CH_3 and CH_2 in lipids and proteins (~1427 cm^{-1}, ~1440 cm^{-1}, ~1400 cm^{-1}, ~1364 cm^{-1}), υPO_2^- (~1248 cm^{-1}, ~1080 cm^{-1}), and carbohydrates (~1160 cm^{-1} , ~1150 cm^{-1}, ~1100, ~1065, and 1024). Sphingomonas-SAM and -HTF differed from other isolates along the second PC-LDA axis, which accounted for 19% of the variation, and was defined by the absorbance in ~1510 cm^{-1}, ~1220 cm^{-1}, ~1155 cm^{-1}, ~1105 cm^{-1}, and ~1050 cm^{-1}. Overall, the SAM isolates orientated around a larger cluster of tropical isolates, with strong separation between the SAM isolates. By contrast, the clustering of HTF isolates would suggest a similar response to OS3, however, the wider cluster of replicate cells for each isolate would further indicate within-population heterogeneity in response.

Supplemental Figures

Supplemental Figure 1a: Characteristic compound absorbance spectra for fatty acids, proteins, and carbohydrates. The bottom spectrum (-) is an example of a mean cluster absorbance spectrum (see main text), while the top spectrum (-) shows its secondary derivative spectrum which is used to resolve overlapping peaks in the data set.

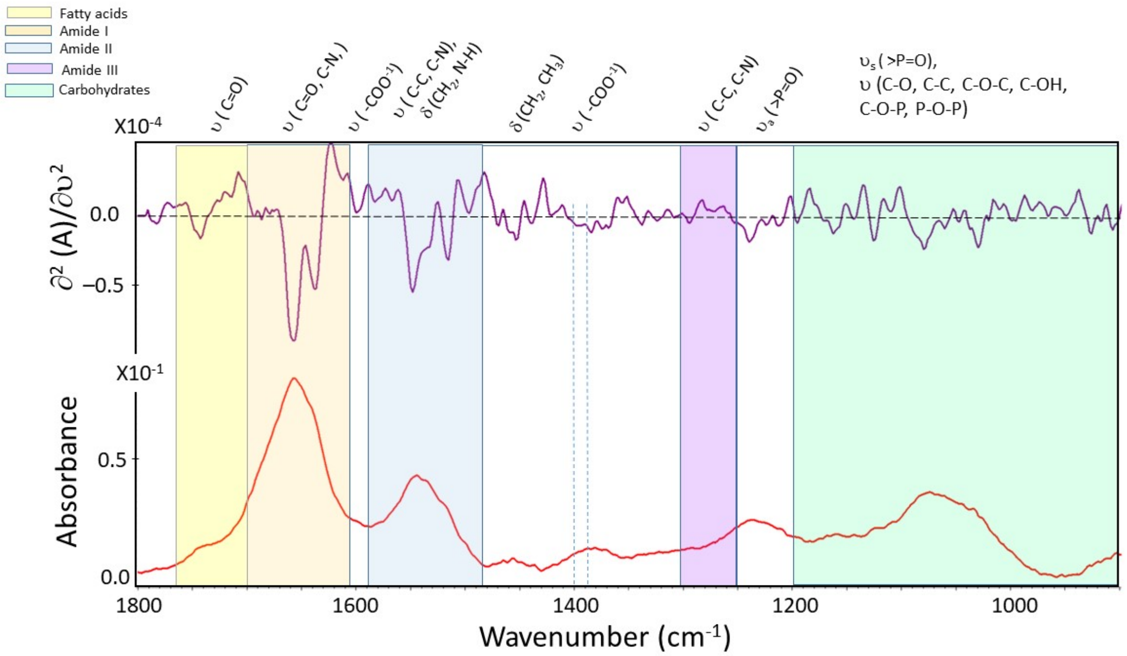

Supplemental Figure 1b: Mean absorbance spectra depicted as the difference between each bacterial isolate as compared with the mean spectrum (plotted as the baseline) at different experimental conditions as follows: (a) control, (b, c) Matric stress-H/-L, (d-f) Osmotic stress 1,2,3. All cluster vector spectra are shifted for ease of visualization.

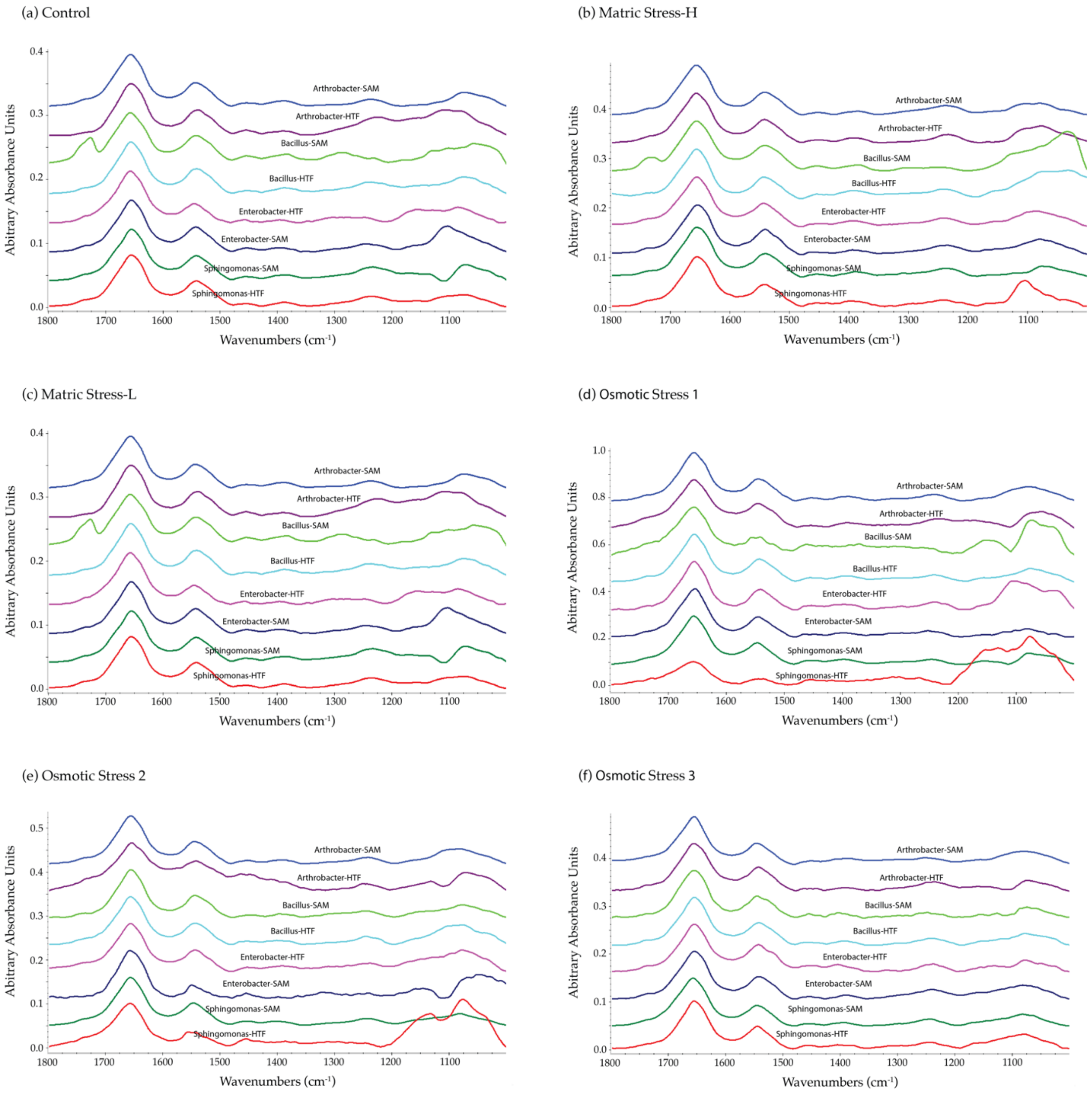

Supplemental Figure 2: The cluster vector spectral plots depicted as the difference between each bacterial isolate as compared with the mean spectrum (plotted as the baseline) at different experimental conditions as follows: (a) control, (b, c) Matric stress-H/-L, (d-f) Osmotic stress 1,2,3. All cluster vector spectra are shifted for ease of visualization.

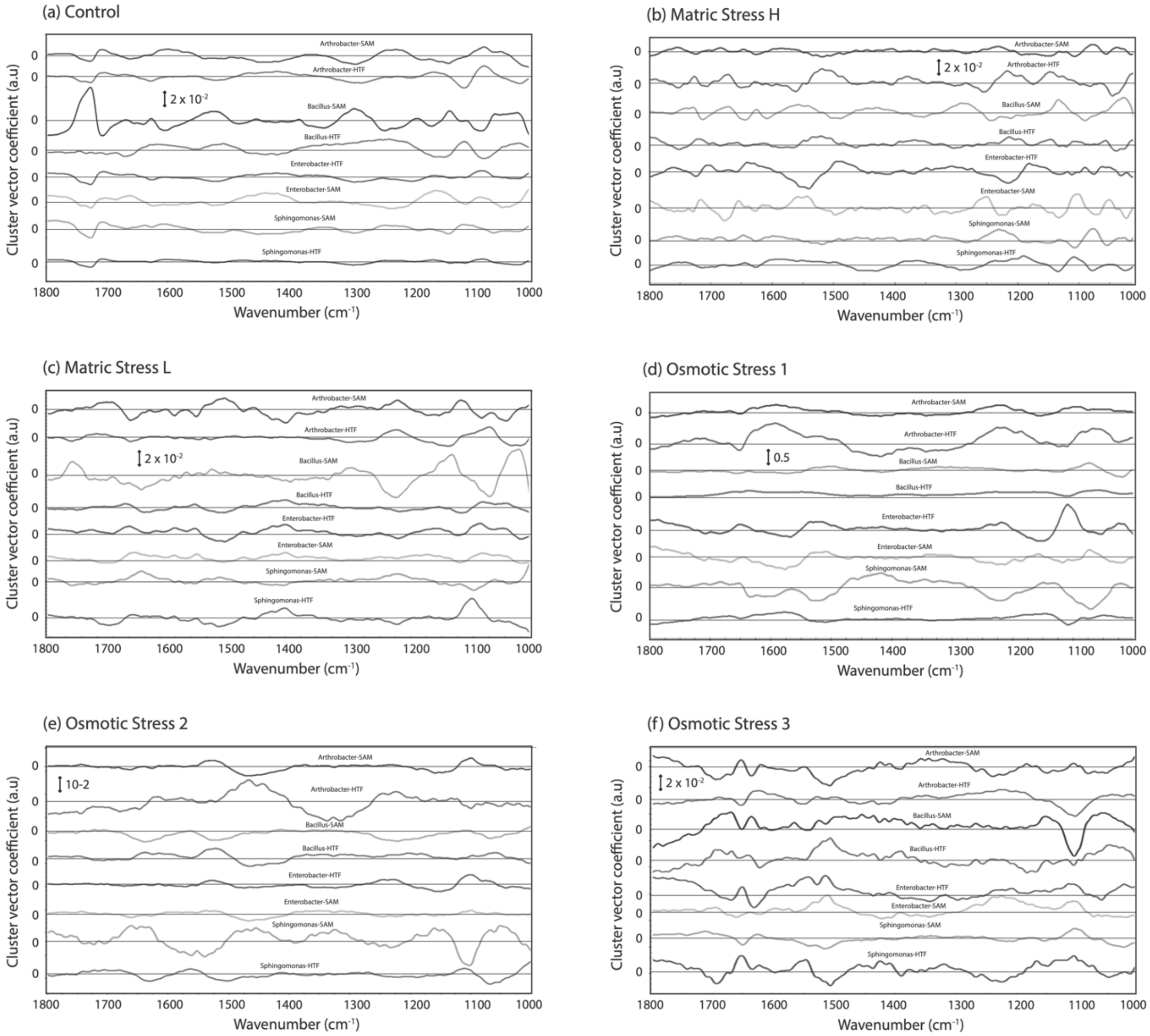

Supplemental Figure 3: Individual 2nd derivatives of the mean spectra for each bacterial isolate. Each panel represents a different experimental condition as follows: (a-b) Matric stress-H/-L, (c, d, e) Osmotic stress 1,2,3., and (f) controls. The negative peaks of the 2nd derivative spectra are used to resolve overlapping peaks.

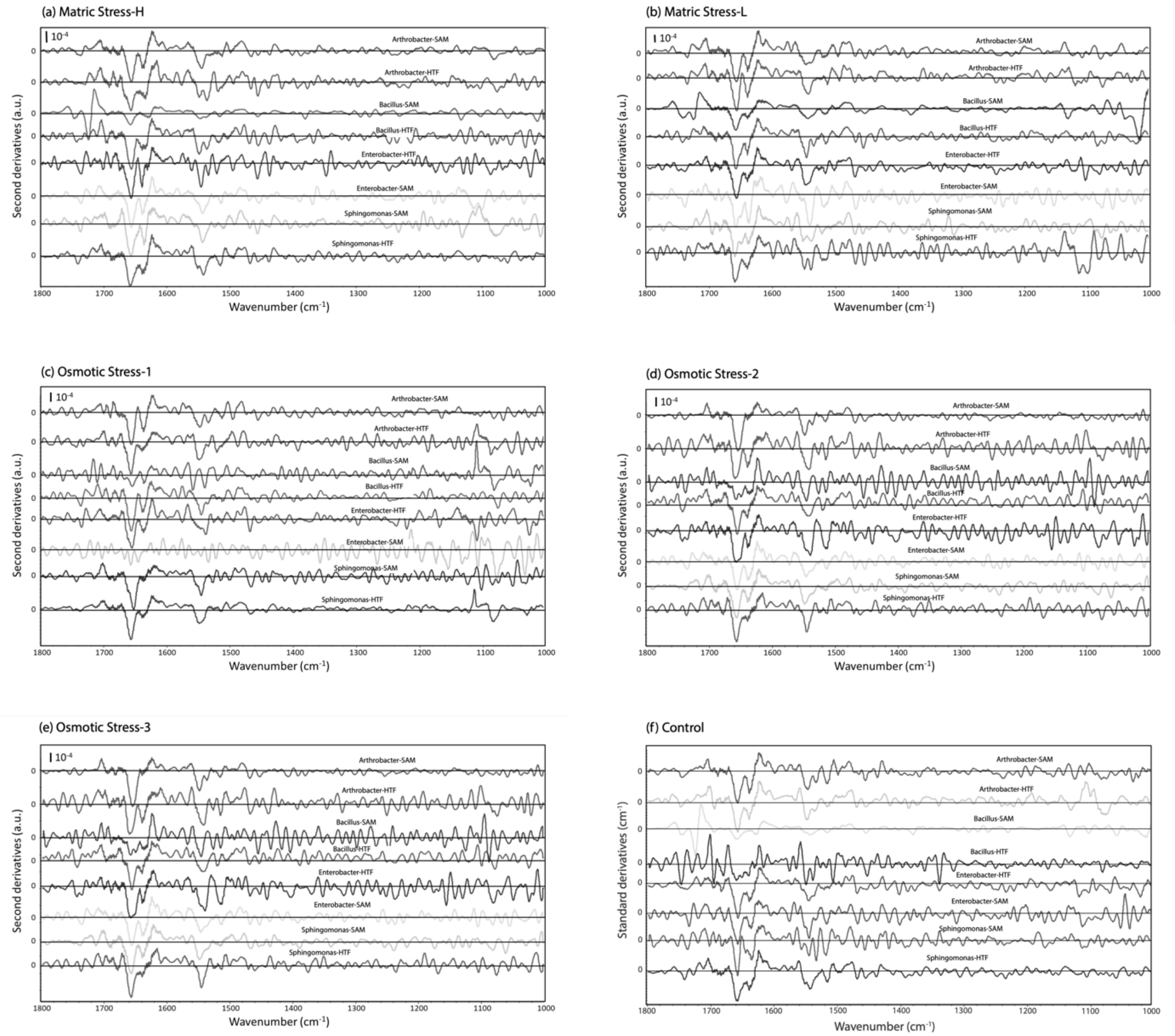

Supplemental Figure 4: Characteristic compound absorbance spectra for osmolytes involved in microbial stress response. Characteristic peaks for each osmolyte are provided in Supplemental Table 3.

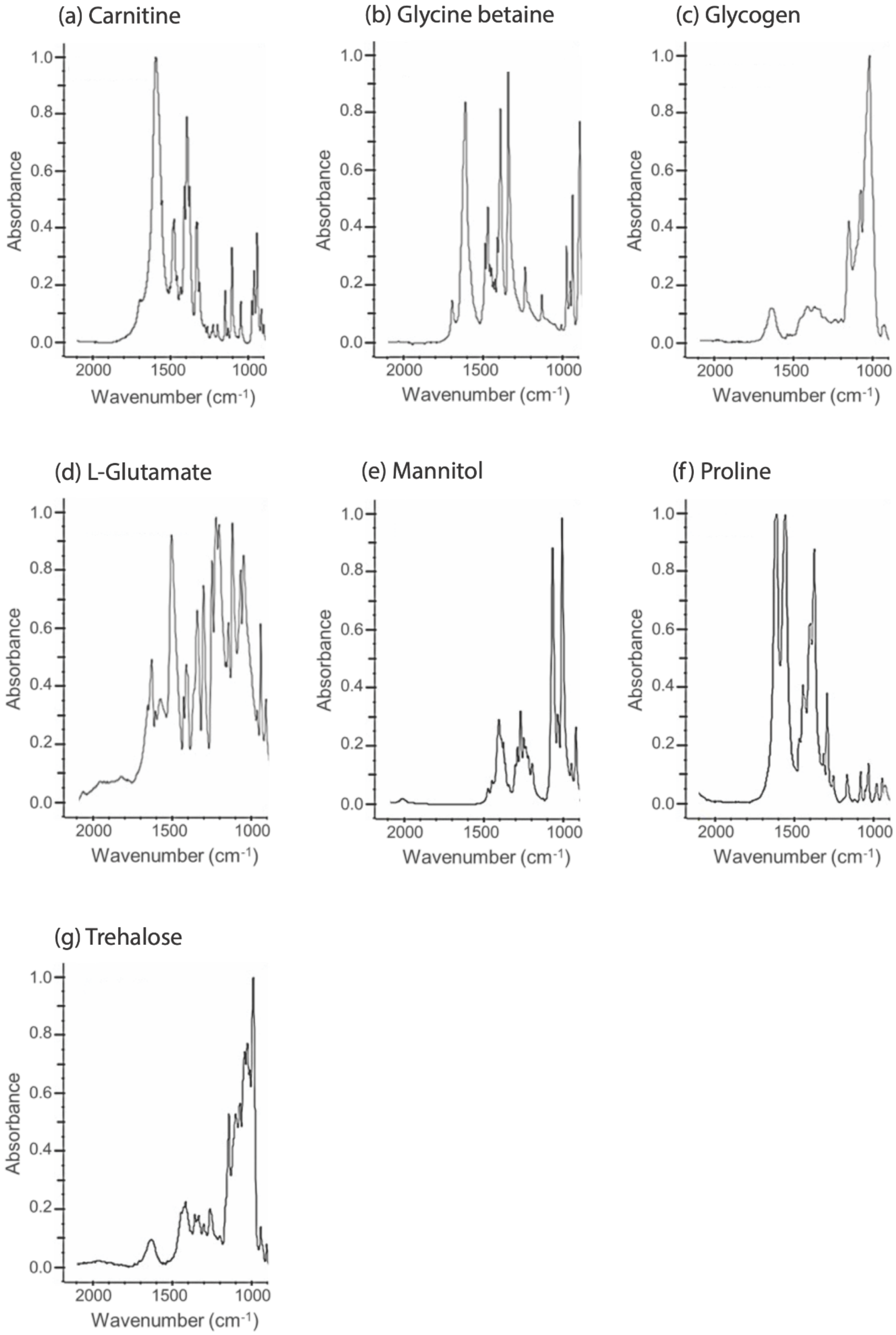

Supplemental Figure 5: Survival rates of all isolates under the MS and OS treatments. Survival is based on live-dead staining and flow cytometry.

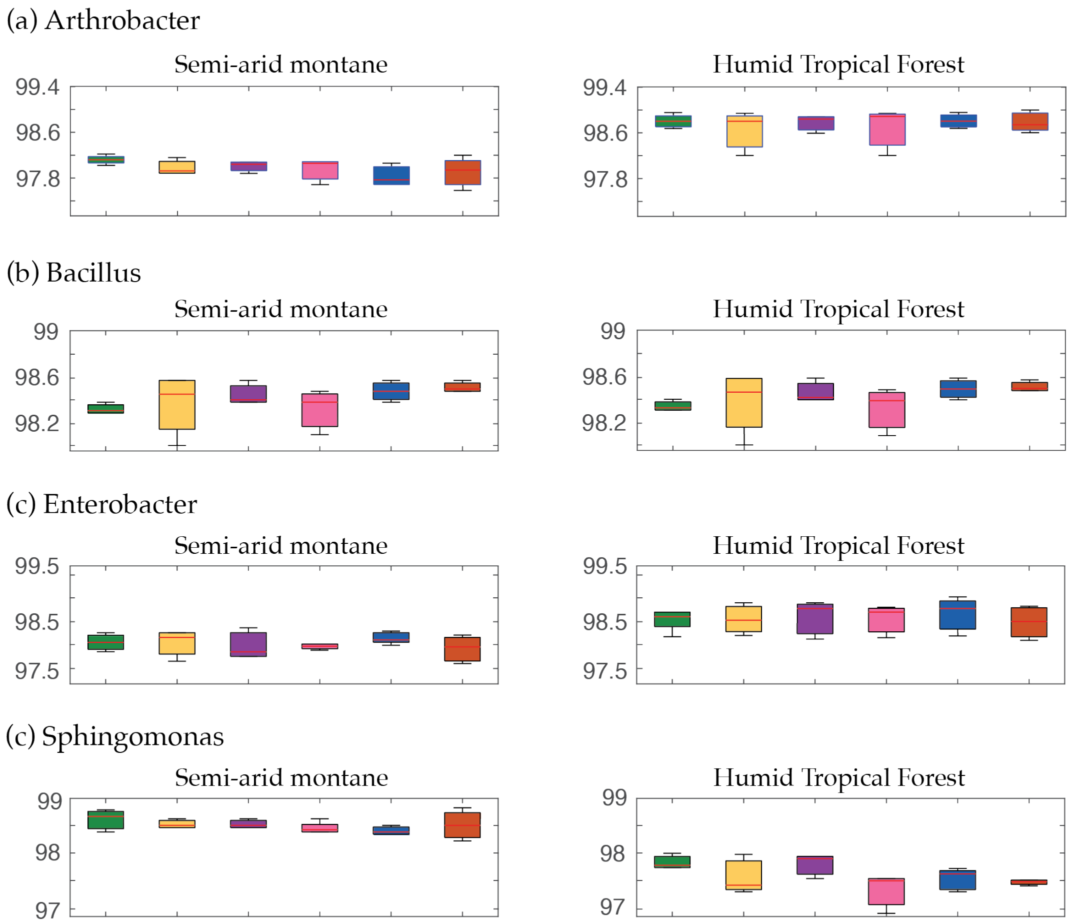

Supplemental Figure 6: The first three PC-LDA loading plots. These plots are based on all relative positioning of all isolates together (i.e., Arthrobacter-SAM, Arthrobacter-HTF, Bacillus-SAM, Bacillus-HTF, Enterobacter-SAM, Enterobacter-HTF, Sphingomonas-AM, and Sphingomonas-HTF) under different experimental conditions as follows: (a) control, (b, c) Matric stress-H/-L, (d-f) Osmotic stress 1,2,3.

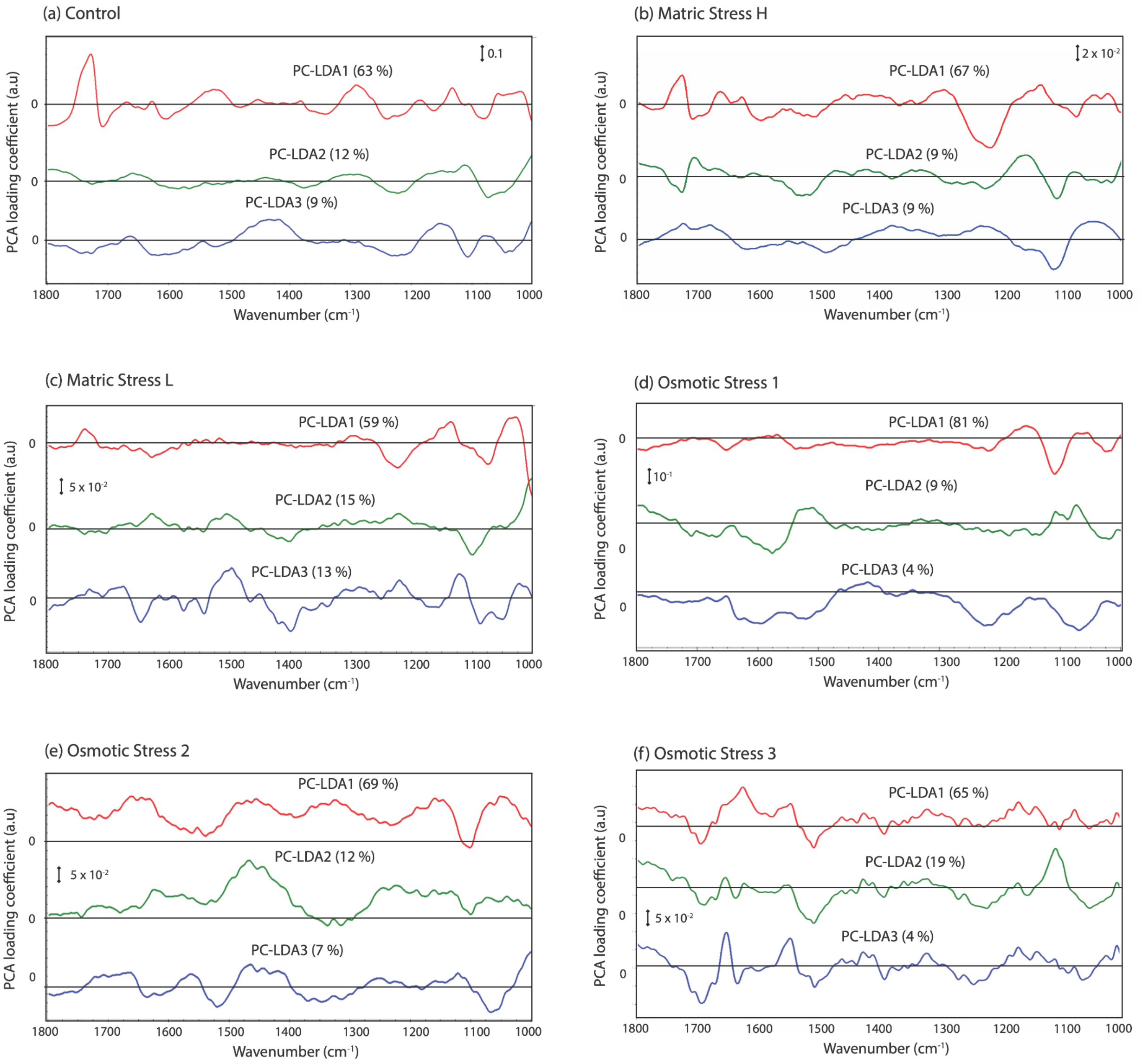

Supplemental Figure 7: Ordination plots across the first three factors based on Principal Component-Linear Discrimination Analysis for each isolate. Each panel, visualized with Varimax Rotation, shows the relative dissimilarity of a single isolate under different experimental conditions of osmotic and matric stress. The panels provide results for bacteria isolated from semi-arid montane (SAM) and humid tropical forest (HTF).

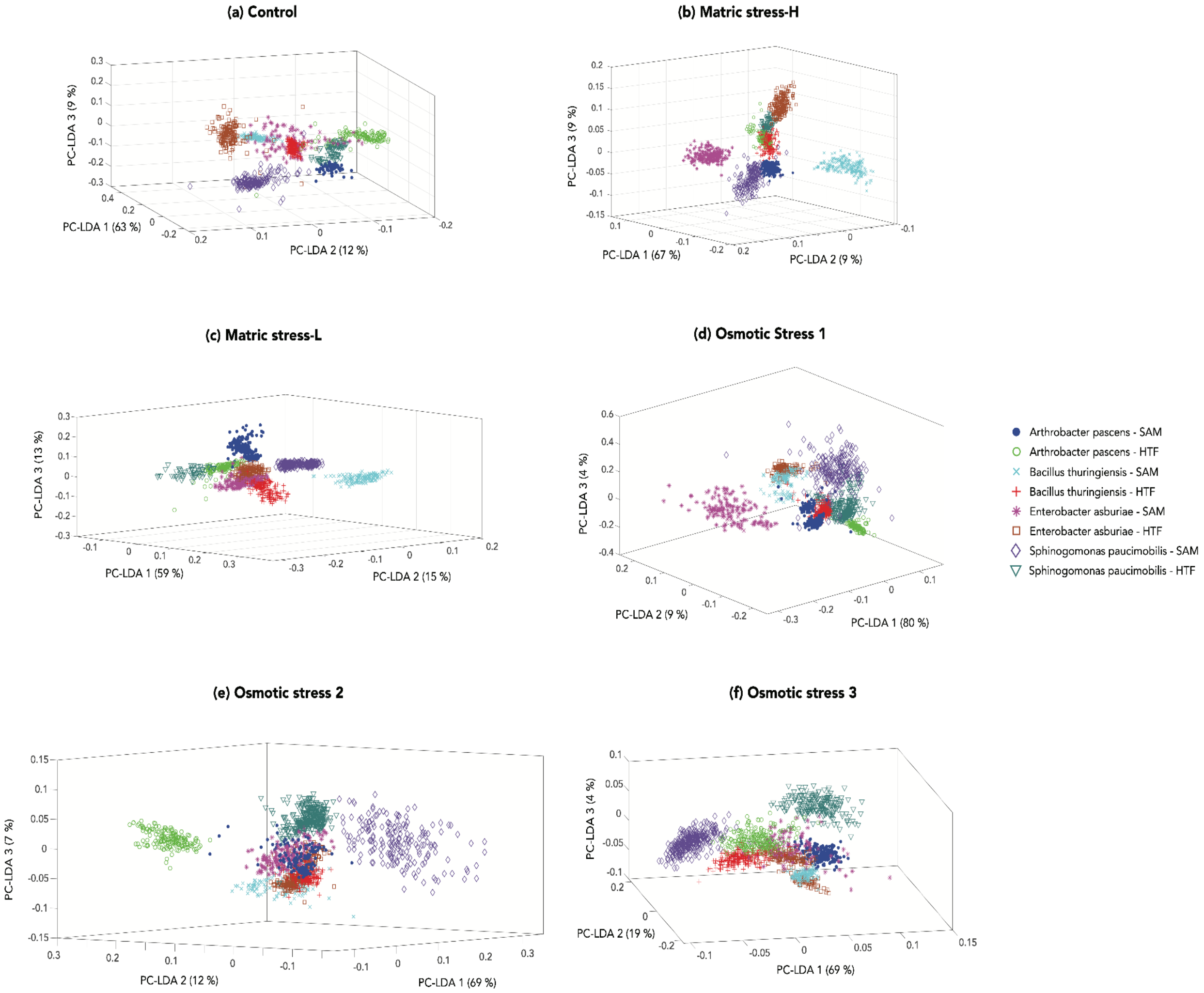

Supplemental Figure 8: Metabolite responses to the matric stress experiments for each isolate. The figure depicts the aggregate shifts in the regions representing lipids and fatty acids (1760 - 1700 cm^{-1}), the phosphate region (1250 - 1200 cm^{-1}), and the broader carbohydrate region (1200 - 1000 cm^{-1}) as shown by the mean relative changes (+/- 10% errors) of the amplitude of secondary derivative absorption peak spectra. These shifts are normalized by the control spectra.

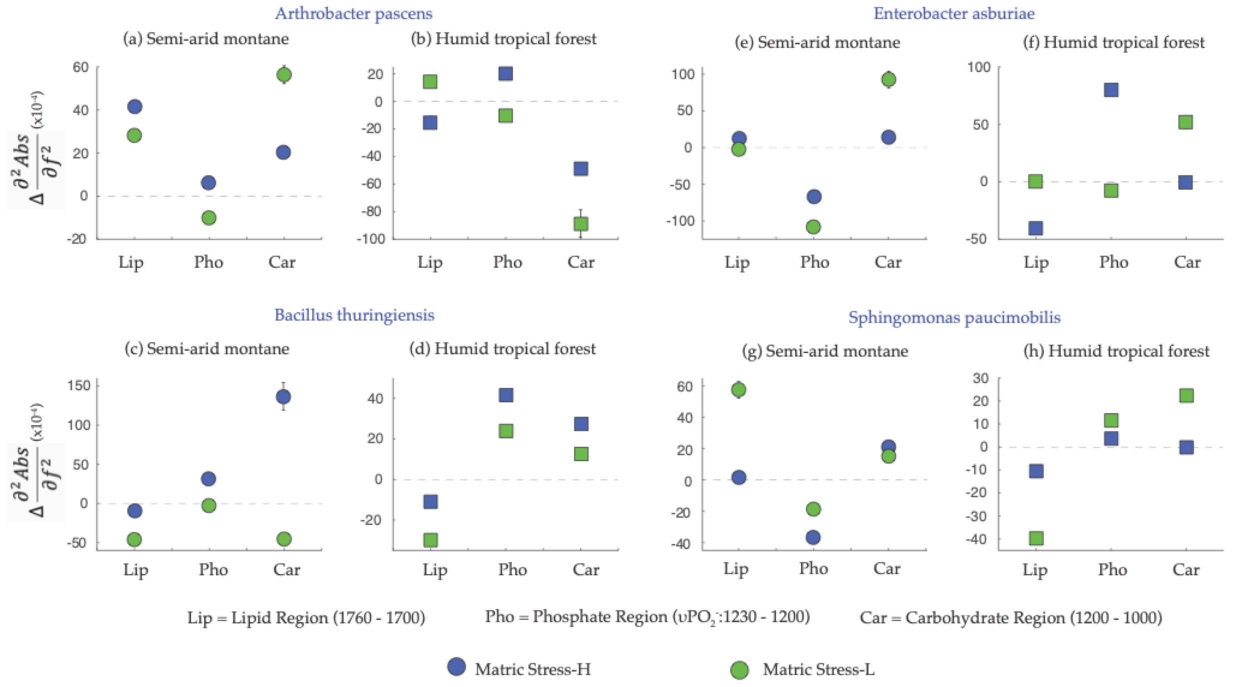

Supplemental Figure 9: Metabolite responses to the osmotic stress experiments for each isolate. The figure depicts the aggregate shifts in the regions representing lipids and fatty acids (1760 - 1700 cm^{-1}), the phosphate region (1250 - 1200 cm^{-1}), and the broader carbohydrate region (1200 - 1000 cm^{-1}) as shown by the mean relative changes (+/- 10% errors) of the amplitude of secondary derivative absorption peak spectra. These shifts are normalized by the control spectra.

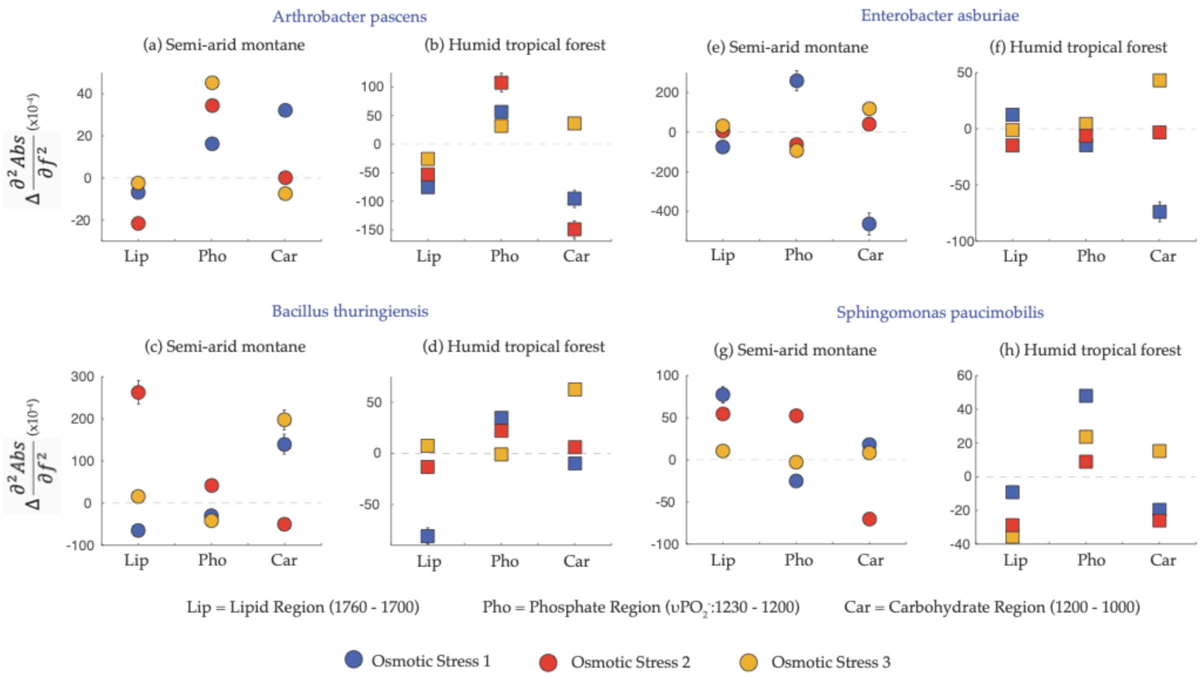

Supplemental Figure 10: Changes in the abundance of (a) storage compounds (e.g., PHA, PHB, glycogen), and (b) osmolytes (e.g., trehalose, glycine betaine, etc.) relative to the control conditions. The data has been aggregated by climate history and experiment (e.g., MS-H, OS-1, etc.). Each color represents a different experiment with SAM: opaque shading, and HTF: transparent shading.

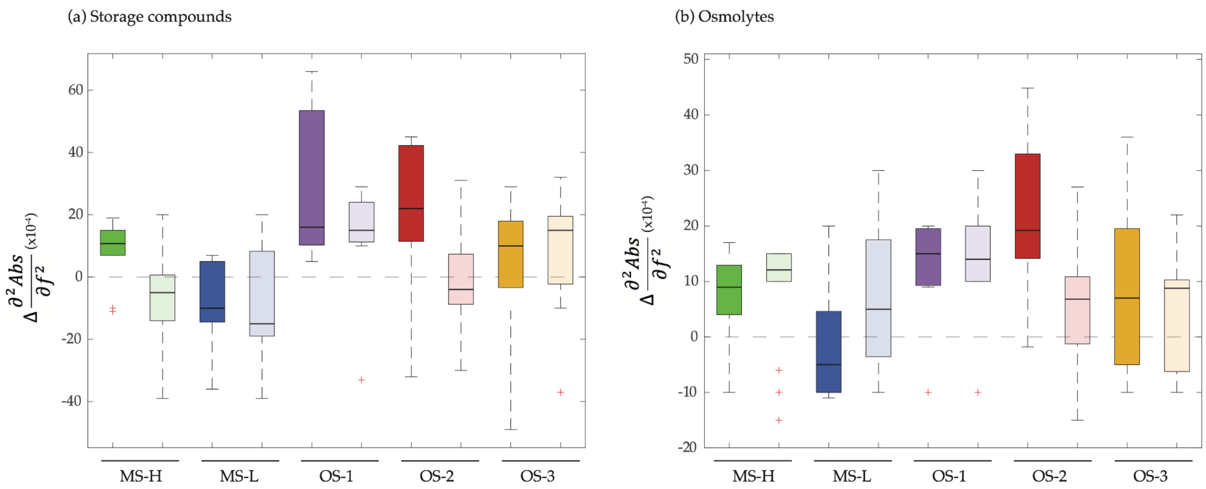

Supplemental Figure 11: The first three PC-LDA loading plots of controls versus matric stress-H versus matric stress-L versus osmotic stress 1 versus osmotic stress 2 versus osmotic stress 3 for each isolate as follows: (a) Arthrobacter-SAM, (b) Arthrobacter-HTF, (c) Bacillus-SAM, (d) Bacillus-HTF, (e) Enterobacter-SAM, (f) Enterobacter-HTF, (g) Sphingomonas-SAM, and (h) Sphingomonas-HTF.

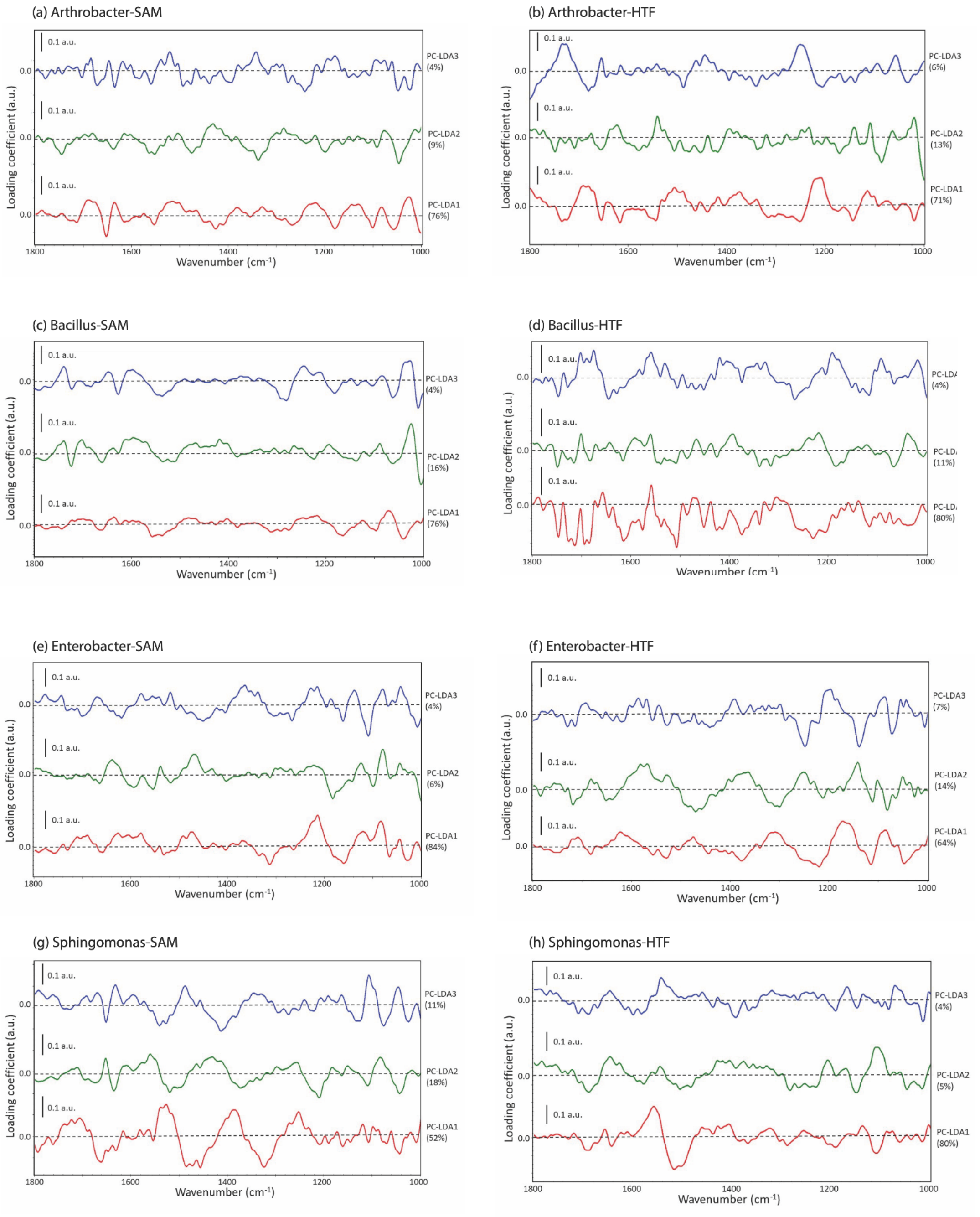

Supplemental Figure 12: The cluster vector spectral plots depicted as the difference between each experimental condition as compared with the mean spectrum (plotted as the baseline) for different bacterial isolates as follows: (a) Arthrobacter-SAM, (b) Arthrobacter-HTF, c) Bacillus-SAM, (d) Bacillus-HTF, (e) Enterobacter-SAM, (f) Enterobacter-HTF, (g) Sphingomonas-AM, (h) versus Sphingomonas-HTF. All cluster vector spectra are plotted with a common scale.

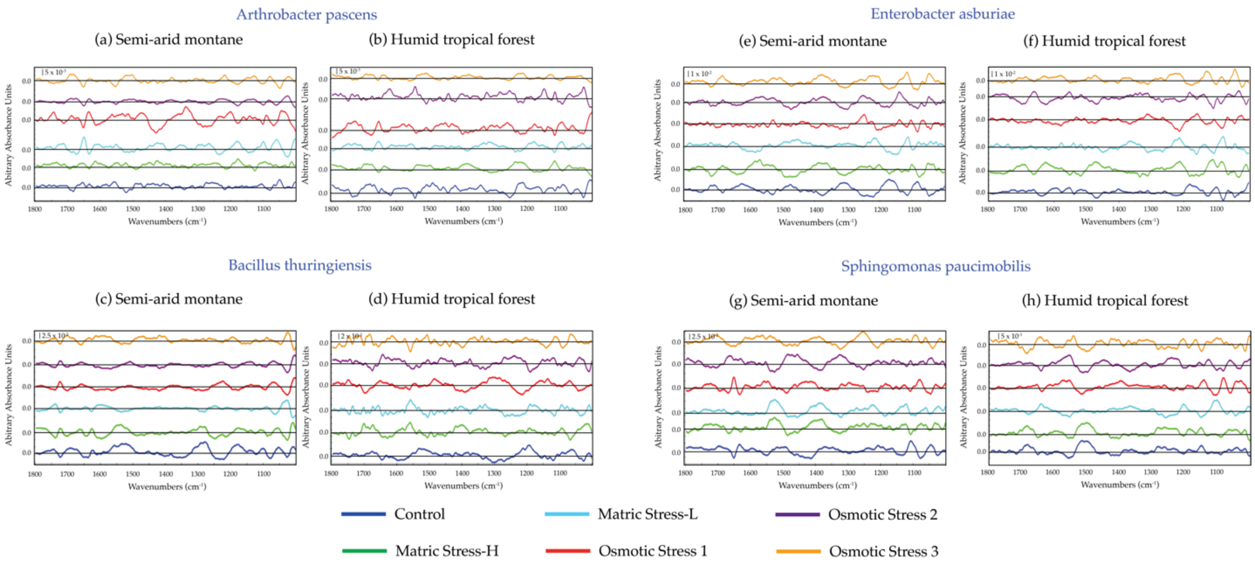

Supplemental Tables

Supplemental Table 1: Isolation media used in the current study.

R2A/ R2G

	R2A (1/10 strength) (g)*	R2G (1/20 strength) (g)*
Protease Peptone	0.05	0.025
Starch	0.05	0.025
Glucose	0.05	0.025
Yeast Extract	0.05	0.025
Casein hydrolysate	0.05	0.025
Dipotassium phosphate	0.3	0.3
Sodium pyruvate	0.3	0.15
Magnesium sulfate	0.024	0.024
Sodium chloride	-	4.5
Agar	14	-
Gelrite	-	12

DNGB

	DNGB (g)*
Difco nutrient broth	0.08
Calcium Chloride	0.07
Gellan gum	8

Inorganic salt starch media

	ISS (g)*
Starch	10
Dipotassium phosphate	1
Magnesium sulfate	1
Sodium chloride	1
Ammonium sulfate	2
Calcum carbonate	2
Ferrous sulfate	0.001
Manganese chloride	0.001
Zinc sulfate	0.001
Agar	20

*1L with ultrapure water (pH = 7.2)

Supplemental Table 2: Isolates used in the current study, location of isolation, and GenBank accession number.

Isolate name	Study ID*	Isolation location	Accession number**
Arthrobacter pascens	Arthrobacter-SAM	Colorado	OQ566217
Arthrobacter pascens	Arthrobacter-HTF	Panama	OQ566218
Bacillus thuringiensis	Bacillus-SAM	Colorado	OQ566219
Bacillus thuringiensis	Bacillus-HTF	Panama	OQ566220
Enterobacter asburiae	Enterobacter-SAM	Colorado	OQ566221
Enterobacter asburiae	Enterobacter-HTF	Panama	OQ566222
Sphingomonas paucimobilis	Sphingomonas-SAM	Colorado	OQ566223
Sphingomonas paucimobilis	Sphingomonas-HTF	Panama	OQ566224

*SAM: Semi-Arid Montane; HTF: Humid Tropical Forest.
**Sequences deposited in GENBANK

Supplemental Table 3: Characteristic absorption peaks for storage and osmotic stress metabolites.

Compounds	IR absorption peaks (cm^{-1})	References
**Energy Storage compounds**		
Polyhydroxyalkanoates (PHA)	~1745-1710 (~1744, ~1740, ~1732, ~1728, ~1720), ~1391, ~1282, ~1262, ~1257, ~1178, ~1128, ~1094, ~1015	Christensen et al 2023; Ojha & Das 2020; Hu et al. 2013; Arcos-Hernandez et al, 2010; Misra et al. 2000;
Polyhydroxybutyrates (PHB)	~1732, ~1379, ~1278, ~1183, ~1132, ~1056	Uchino et al. 2007; Trakunjae et al. 2021
Glycogen	~1150, ~1082, ~1049, ~1026	Fabienne Quilès et al. 2012; Holman et al 2009; König et al. 1982
Polyphosphate	~1275-1260 (PO_2 groups)	Zhang et al. 2016
**Osmolytes**		
Trehalose ($C_{12}H_{22}O_{11}$)	~1150-1000 (~1150, ~1130, ~1100, ~1085, ~1062, ~1031, ~1016, ~1000) (trehalose moieties)	Caccamo et al. 2022; Kuroiwa et al. 2015; Akao et al. 20019
Mannitol ($C_6H_{14}O_6$)	~1450, ~1420, ~1280, ~1082, ~1019	Bayón & Rojas, 2017
Ectoine ($C_6H_{10}N_2O_2$)	~1602, ~1388, ~1308, ~1292, ~1270	Golovina et al. 2014; Anburajan et al. 20
Proline ($C_5H_9NO_2$)	~1625, ~1616, ~1565, ~1409, ~1380,	Liu et al. 2021
Glutamate ($C_5H_9NO_4$)	~1596, ~1562, ~1450, ~1333	Parikh et al. 2011
Glycine betaine ($C_5H_{11}NO_2$)	~1493, ~1481, ~1453, ~1400, ~1335, ~1234, ~1122	Kirschenbaum, 1963
Carnitine ($C_7H_{15}NO_3$)	~1731, ~1724, ~1685, ~1580, ~1483, ~1411, ~1383, ~968	Acosta et al. 2020

Supplemental Table 4: Characteristic key absorption peak frequencies in the mid-infrared spectral range from 1800 to 1000 cm^{-1} for Arthrobacter-SAM under different types of stress. The prominent adsorption peak frequencies for the control cells are provided in black font and the changes under the stress are recorded in the adjacent columns. Where no change is recorded, the peak from the control did not appear under the stress condition. Peak wavenumbers in orange indicate a new peak not observed under control conditions but might appear in one, or more, of the stress conditions. Key: ↑: peak increased in relative intensity under stress. ↓ peak decreased in relative intensity under stress. - the peak was present at the same relative intensity. ↑→ / ↓→: increasing/ decreasing peak intensity accompanied by redshift. ←↑/ ←↓: increasing/ decreasing peak intensity accompanied by a blue shift.

(a) Important carbonyl (C=O) bands of lipids & fatty acids

Peak Frequency (cm^{-1})	Assignment	Prominent adsorption peaks in control & stress experiments					
		Ctl.	MS1	MS2	OS1	OS2	OS3
1750 – 1700	υ carbonyl C=O	1752		↑			
		1746	↓→ 1742	↓	↑	↓	↓
~1748 – 1740	υ C=O of ester (in PHA, confirmed by the presence of C-O of alkoxy group at 1311 cm^{-1}, and the CH=CH2 at 1027 cm^{-1} (Nwinyi et al., 2017))	1740				↑	↑
		1736	↑	↑		↑	
~1734	υ C=O of saturated long-chain esters or ether esters						

(b) Important amide I and amide II bands of proteins

**Peak Frequency (cm^{-1})**	**Assignment**	**Prominent adsorption peaks in control & stress experiments**					
		**Ctl.**	**MS1**	**MS2**	**OS1**	**OS2**	**OS3**
		1695					↑
~1690 – 1610	υC=O and υC-N of Amide I band components	1690		↑	↑	↑	
		1683					↑
		1676	↓→				
		1659	↑→ 1657	↑→ 1657	↑→ 1657	↑→ 1657	↑→ 1655
		1639	↑→ 1638	↑	↑→ 1638	↑	↓
~1580 – 1470<br>~1577<br>~1549<br>~1538	υC-N, υC-C, and δN-H of Amide II band components	1548	↑→ 1545	↓	↑	↑	↑→ 1545
		1535			← 1535	↓	← 1535
		1516	↓→ 1514	↓	↓→ 1514	↓→ 1514	↓→ 1514
~1518 - 1495	Aromatic rings (including Tyr & Phe)	1499	↓	↑→ 1496	↑→ 1496	↑→ 1496	

(c) Important complex infrared absorption bands of lipids, fatty acids and proteins

Peak Frequency (cm^{-1})	Assignment	Prominent adsorption peaks in control & stress experiments					
		Ctl.	MS1	MS2	OS1	OS2	OS3
~1475 – 1370<br>~1470<br>~1456<br>~1439	δC-H of CH_3, CH_2<br>C-H def of $>CH_2$	1467	↑	↑	↓		↑
		1456	↓	↱ 1453	↑	↳ 1454	↲ 1457
		1437	↓	↲ 1441	↲ 1440		
		1421					
		1412		↑			
		1407					↑
~1440 – 1395	C-O of carboxylic acid or<br>-COO of carboxylate	1400	↑	↑	↰ 1407	↑	
		1389	—	↑	↑	↑	
		1389		↑			
		1382					
~1370 +/– 20	Bending (δ, ρ, ω, τ) of OH of primary & secondary alcohols	1371		↓	↑	↑	← 1373

Table c cont.

Peak Frequency (cm^{-1})	Assignment	Prominent adsorption peaks in control & stress experiments					
		Ctl.	MS1	MS2	OS1	OS2	OS3
~1310 – 1240	Amide III components of protein<br>1300 cm^{-1} α-helix<br>1285 & 1245 cm^{-1} random coil<br>1265 & 1235 cm^{-1} β-turn spectrum	1317			↑		
		1315		↑			
		1312	↑			↑	
		1308					
		1305				↑	
		1304			↑		
		1302					↑
		1286		↑		↑	↑
		1261		↑			
		1245		↑			
		1238		↑			

(d) Important infrared absorption bands of carbohydrates

Peak Frequency (cm^{-1})	Assignment	Prominent adsorption peaks in control & stress experiments					
		Ctl.	MS1	MS2	OS1	OS2	OS3
		1253			↑ 1255		↑
	υ_{as} P-O of >PO_2^- phosphodiester	1243	↑				
1250 – 1220	1210 -1215 cm^{-1} for Z-form<br>1222 -1225 cm^{-1} for B-form	1238	↓		↓		
	1240 -1250 cm^{-1} for A-form	1235	↑				↑
		1223		↑ 1221	↑ 1230		
		1218					↑
	δ C-H of CH_2 coupled with	1211		↓	↓ 1208	↑	
	C-O modes	1195	↓		↓ 1186	↓	
1240 – 1171		1181					↑
	υ of C-O-C linkages -	1170		↑			
	1160 cm^{-1} carbohydrate rich	1168	↑			↑	
	bands (Nichols, 1985)	1160	↓		↑ 1166		
		1159		↑			
		1144				↓	
		1133	↑				
1140 – 1070	υ_{as} C-O of saturated unbranched ethers in carbohydrates -	1125	↓	↓ 1120			
		1121	↑				↑
1300 – 1200/ 1050 - 1000	υ_{as} C-O of mixed (alkyl/ aryl) ethers in carbohydrates -	1113			↑	↑	↑
		1110					
1200 – 1000	υ_s C-O, C-C, and C-O-H, C-O-C def of carbohydrates	1103			↑		↑

(d) Important infrared absorption bands of carbohydrates (cont.)

Peak Frequency (cm^{-1})	Assignment	Prominent adsorption peaks in control & stress experiments					
		Ctl.	MS1	MS2	OS1	OS2	OS3
1090 – 1085	υ_{as} P-O of $>PO_2^-$	1099		↑	↑	↑	
		1090					
		1083	↓		↑		↓
1150 – 1070	υ_{as} C-O of secondary hydroxyl - 1150: carbohydrate of bacterial gum (Nichols, 1985)	1081	↑	↑		↑	↑
		1076	↓		↓		↓
		1069			↑		
		1061	↱ 1060	↱ 1060			
		1059			↑		↑
		1054	↑				
		1045	↓				
1075 – 1000	υ_{as} C-O of primary hydroxyl - of carbohydrates	1040	↑				
		1038		↑			↑
		1034	↓			↳ 1033	
		1032		↑			
		1030	↑				
		1028					↴ 1029
		1024					
		1023		↑			
		1021	↑		↑		
		1018			↑		↑
		1014				↑	
		1005					↑

Supplemental Table 5: Characteristic key absorption frequencies in the mid-infrared spectral range from 1800 to 1000 cm^{-1} for Arthrobacter-HTF under different types of stress. Refer to supplemental table 4 for additional information on adsorption peak assignment, and a table key.

(a) Lipid & fatty acid carbonyl (C=O) bonds region (~1780 - 1700 cm-1).

Peak Frequency (cm^{-1})	Assignment	Prominent adsorption peaks in control & stress experiments					
		Ctl.	MS1	MS2	OS1	OS2	OS3
1750 – 1700	υ carbonyl C=O	1750			↑	↑	
		1748					
~1748 – 1740	υ C=O of ester (in PHA,	1745	↓	↓	↑		
		1743			↑		↑
		1738			↑	↑	
		1736	↑	↑			
~1734	υ C=O of saturated long-chain esters or ether esters	1733			↑		
		1731	↑				
~1722	υ C=O of saturated aldehydes	1724				↑	↑

(b) Important amide I and amide II bands of proteins

Peak Frequency (cm^{-1})	Assignment	Prominent adsorption peaks in control & stress experiments					
		Ctl.	MS1	MS2	OS1	OS2	OS3
1715 – 1680	υ-carbonyl C=O	1695			↱ 1693	↴ 1699	↱ 1691
		1688		↑			
~1690 – 1610	υC=O and υC-N of Amide I band components	1682			↑		
		1675		↑			↑
		1657	↱ 1656	↳ 1656	↑	↓	↴ 1658
		1640	↓	↑	↓	↓	—
		1635	↓				
		1616				↑	
		1577	↑			↓	
		1569					↑
		1560				↑	
~1580 – 1470		1558					
~1577	υC-N, υ C-C, and δ N-H of Amide II band components	1549	↓		↓		↑
~1549		1541				↑	
~1538		1537	↑	↱ 1553	↱ 1553	↓	
~1518 - 1495		1527					↑
		1516	↲ 1520	↱ 1514	↲ 1520	↱ 1514	↑
~1520 – 1490	Aromatic rings (Incl. Tyr and Phe)	1495	↑	↑		↑	

(c) Important complex infrared absorption bands of lipids, fatty acids and proteins

Peak Frequency (cm^{-1})	Assignment	Prominent adsorption peaks in control & stress experiments					
		Ctl.	MS1	MS2	OS1	OS2	OS3
		1470	—	—	↓	↱ 1469	↱ 1469
		1456	↰ 1457	↓	↓		↓
~1475 – 1370		1440	↑			↑	
~1470	δC-H of CH_3, CH_2	1438		↑			↑
~1456	C-H def of $>CH_2$	1436			↑		
~1439	C-O of carboxylic acid or	1426				↑	↑
~1440 – 1395	-COO of carboxylate	1418	↰ 1420		↓		
		1426			↑	↑	↑
		1403		↑		↑	↑
		1418					
		1393	↑		↑	↑	↑
	Bending (δ, ρ, ω, τ) of OH of primary & secondary alcohols	1388		↲ 1390			
~1370 +/– 20	(1370 cm-1 indicate carbohydrate-rich bands)	1374	↰ 1378	↱ 1373	↓	↑	
		1354		↑	↑	↑	
		1347					↑
		1340			↓		
		1332		↑	↑	↑	↑
		1311	—	↲ 1315	↰ 1317		
		1306				↑	
		1300			↑		
	Amide III components of protein	1297	↑				↑
	1300 cm^{-1} α-helix	1287		↑			
~1310 – 1240	1285 & 1245 cm^{-1} random coil	1285				↰ 1287	
	1265 & 1235 cm^{-1} β-turn spectrum	1281					↑
		1274			↑		
		1264			↑		
		1252	↑			↑	
		1251					↑
		1241		↰ 1246	↱ 1240	↰ 1246	

(d) Important infrared absorption bands of carbohydrates

Peak Frequency (cm^{-1})	Assignment	Prominent adsorption peaks in control & stress experiments					
		Ctl.	MS1	MS2	OS1	OS2	OS3
1200 – 1000	υ_s C-O, C-C, and C-O-H, C-O-C def of carbohydrates	1232		↑	↱ 1238		↑
1300 – 1200/ 1050 - 1000	υ_{as} C-O of mixed (alkyl/ aryl) ethers in carbohydrates - 1240: carbohydrate rich bands	1222		↳ 1218	↳ 1225		↱ 1220
		1217			↑	↑	
1250 – 1220	υ_{as} P-O of $>PO_2^-$ phosphodiester	1208					
		1201			↑		↑
		1187			↑	↑	↑
		1179			↑		
	δ C-H of CH_2 coupled with C-O modes	1174	↑				
1240 – 1171		1168		↑		↑	
	υ of C-O-C linkages - 1160: carbohydrate rich bands (Nichols, 1985)	1165	↑				↑
		1159			↑		↑
		1155	↑				
		1154				↑	
		1146			↑		↑
		1132			↓	↑	
		1126					↑
		1122	↑	↑			
1140 – 1070	υ_{as} C-O of saturated unbranched ethers in carbohydrates -	1114	↑	↑			
		1110					↑
		1104	↑	↑			
1090 – 1085	υ_{as} P-O of $>PO_2^-$	1087			↑	↓	
1150 – 1070	υ_{as} C-O of secondary hydroxyl - 1150: carbohydrate of bacterial gum (Nichols, 1985)	1080					↑
		1077	↓	↑	↓		
		1075				↓	↓

(d) Important infrared absorption bands of carbohydrates

Peak Frequency (cm^{-1})	Assignment	Prominent adsorption peaks in control & stress experiments					
		Ctl.	MS1	MS2	OS1	OS2	OS3
1075 – 1000	υ_{as} C-O of primary hydroxyl - of carbohydrates	1067	↑				
		1062		↑		↓	↑→ 1061
		1055			↑	↑	
		1037					
		1032	↑				↑
		1030	↑				
		1028					
		1021		↑	↑	↑	↑
		1011	↑		↑	↑	
		1005					↑

Supplemental Table 6: Characteristic key absorption frequencies in the mid-infrared spectral range from 1800 to 1000 cm^{-1} for Bacillus-SAM under different types of stress. Refer to supplemental table 4 for additional information on adsorption peak assignment, and a table key.

(a) Important carbonyl (C=O) bands of lipids & fatty acids

**Peak Frequency (cm^{-1})**	**Assignment**	**Prominent adsorption peaks in control & stress experiments**					
		**Ctl.**	**MS1**	**MS2**	**OS1**	**OS2**	**OS3**
1715 – 1680	υ-carbonyl C=O of nucleic acids:	1690			↑	↑	
		1669				↑	
		1660	↑	↑→ 1659	←↑ 1665	↑→ 1657	
		1658					↑
		1650					↑
		1640		↑			
~1690 – 1610	υC=O and υC-N of Amide I band components	1638				↑	↑
		1632					↑
		1629	↓				↓
		1594			↑		
		1578			↑	↑	
		1560			↑	↓	↓
~1580 – 1470<br>~1577<br>~1549<br>~1538<br>~1518 - 1495	υC-N, υ C-C, and δ N-H of Amide II band components	1543	↑	↓	↑→ 1541	↑→ 1541	
		1521				↑	
		1517	↑	↓	←↑ 1619		↑
		1511				↑	
~1520 – 1490	Aromatic rings (Incl. Tyr and Phe)	1498				↑	↓

(b Important amide I and amide II bands of proteins

Peak Frequency (cm^{-1})	Assignment	Prominent adsorption peaks in control & stress experiments					
		Ctl.	MS1	MS2	OS1	OS2	OS3
1715 – 1680	υ-carbonyl C=O of nucleic acids:	1690			↑	↑	
		1669				↑	
		1660	↑	↳ 1659	↲ 1665	↳ 1657	
		1658					↑
		1650					↑
		1640		↑			
~1690 – 1610	υC=O and υC-N of Amide I band components	1638				↑	↑
		1632					↑
		1629	↓				↓
		1594			↑		
		1578			↑	↑	
		1560			↑	↓	↓
~1580 – 1470	υC-N, υ C-C, and δ N-H of Amide II band components	1543	↑	↓	↳ 1541	↳ 1541	
~1577		1521				↑	
~1549		1517	↑	↓	↲ 1619		↑
~1538		1511				↑	
~1518 - 1495							
~1520 – 1490	Aromatic rings (Incl. Tyr and Phe)	1498				↑	↓

(c) Important complex infrared absorption bands of lipids, fatty acids and proteins

Peak Frequency (cm^{-1})	Assignment	Prominent adsorption peaks in control & stress experiments					
		Ctl.	MS1	MS2	OS1	OS2	OS3
		1473				↑	↓
~1475 – 1370		1468	↓	↑		↑	↑
~1470	δC-H of CH_3, CH_2	1456	↑	↓	↓	↑	↑
~1456	C-H def of >CH_2	1440	↑				
~1439		1425			↑	↑	
~1440 – 1395	C-O of carboxylic acid or -COO of carboxylate	1417					—
		1413				↑	
	Bending (δ, ρ, ω, τ) of OH of primary & secondary alcohols	1381	↑	↓	↑	↓	↑
~1370 +/– 20	(1370 cm-1 indicate carbohydrate-rich bands)	1371			↑	↑	↓
		1357				↑	
		1348				↑	
		1346			↑		↓
		1338				↑	
		1332			↑		↓
		1327				↑	
		1314				↑	
		1309	↰ 1311	↱ 1307	—		↓
	Amide III components of protein	1293	↰ 1292	↓	↑		↓
~1310 – 1240	1300 cm^{-1} α-helix<br>1285 & 1245 cm^{-1} random coil<br>1265 & 1235 cm^{-1} β-turn spectrum	1278	↑				
		1265			↓	↑	
		1262	↓	↓			↓
		1255			↓	↑	
		1246				↑	↓

(d) Important infrared absorption bands of carbohydrates

Peak Frequency (cm^{-1})	Assignment	Prominent adsorption peaks in control & stress experiments					
		Ctl.	MS1	MS2	OS1	OS2	OS3
1250 – 1220	υ_{as} P-O of >PO_2^- phosphodiester	1230	↑	↓		↓	—
		1222			↑		
1240 – 1171	δ C-H of CH_2 coupled with C-O modes	1209				↑	
	υ of C-O-C linkages - 1160: carbohydrate rich bands	1203			↑		
		1194				↑	
		1183	↑				↑
		1177				↑	
		1172		↑	↑		↓
		1163				↑	
		1154	↑		↑	↑	↓
		1142				↑	
1140 – 1070	υ_{as} C-O of saturated unbranched ethers in carbohydrates -	1138			↑		
		1133	↑	↓		↑	↑
1200 – 1000	υ_s C-O, C-C, and C-O-H, C-O-C def of carbohydrates	1129			↑		
1300 – 1200/ 1050 - 1000	υ_{as} C-O of mixed (alkyl/ aryl) ethers in carbohydrates - 1240: carbohydrate rich bands						

(d) Important infrared absorption bands of carbohydrates

Peak Frequency (cm^{-1})	Assignment	Prominent adsorption peaks in control & stress experiments					
		Ctl.	MS1	MS2	OS1	OS2	OS3
		1090				↑	
		1083			↑		
1150 – 1070	υ_{as} C-O of secondary hydroxyl - 1150: carbohydrate of bacterial gum	1080		↑			
		1073				↑	
		1069			↑		
1090 – 1085	υ_{as} P-O of $>PO_2^-$	1059		↓	↑		↓
		1043	↓	↑			—
		1035			↑	↑	
1075 – 1000	υ_{as} C-O of primary hydroxyl - of carbohydrates	1030	↑		↑		↑
		1025				↑	
		1013	↑	↑	↑		↑

Supplemental Table 7: Characteristic key absorption frequencies in the mid-infrared spectral range from 1800 to 1000 cm^{-1} for Bacillus-HTF under different types of stress. Refer to supplemental table 4 for additional information on adsorption peak assignment, and a table key.

(a) Important carbonyl (C=O) bands of lipids & fatty acids

Peak Frequency (cm^{-1})	Assignment	Prominent adsorption peaks in control & stress experiments					
		Ctl.	MS1	MS2	OS1	OS2	OS3
1750 – 1700	υ carbonyl C=O	1749			↓	↑	
~1748 – 1740	υ C=O of ester	1747	↓	↓		↓	↓
~1734	υ C=O of saturated long-chain esters or ether esters	1743		↑			↑
~1722	υ C=O of saturated aldehydes	1737	↑		↑	↑	
		1733		↑	↓		
~1715	υ C=O of saturated aliphatic ketones	1731	↓	↓			↓
		1716					↑
1713 - 1709	υ C=O of saturated aliphatic carboxylic acids	1714	↓	↓	↓		↓
		1702			↑		

(b) Important amide I and amide II bands of proteins

Peak Frequency (cm^{-1})	Assignment	Prominent adsorption peaks in control & stress experiments					
		Ctl.	MS1	MS2	OS1	OS2	OS3
1715 – 1680	υ carbonyl C=O	1695	↑				
		1681	↓				
		1660					↓
~1690 – 1610	υC=O and υC-N of Amide I band components	1652	←↑ 1656	←↑ 1658	←↑ 1657	↑→ 1651	↓→ 1651
		1641	↓	↑	↑→ 1639	↑→ 1639	—
		1634	↑				
		1630					
		1616					
		1576					
		1565					
~1580 – 1470	υC-N, υ C-C, and δ N-H of Amide II band components	1550	↓→ 1549	↓→ 1546	↓→ 1546	↓→ 1549	
~1577		1539	↓	↓→ 1535		↓	
~1549							
~1538		1527					
~1518 - 1495		1518	—	↑→ 1516		↑→ 1516	←↑ 1521
~1520 – 1490	Aromatic rings (Incl. Tyr and Phe)	1508					↑
		1496			↑		

(c) Important complex infrared absorption bands of lipids, fatty acids and proteins

Peak Frequency (cm^{-1})	Assignment	Prominent adsorption peaks in control & stress experiments: Ctl.	MS1	MS2	OS1	OS2	OS3
		1469	↑	↑	—	↑	↑
~1475 – 1370		1453	⤵ 1456	⤵ 1456	⤵ 1456	↓	↓
~1470<br>~1456	δC-H of CH_3, CH_2<br>C-H def of >CH_2	1442					↑
~1439		1438	↑		↑	↑	
	C-O of carboxylic acid or<br>-COO of carboxylate	1433					↑
~1440 – 1395		1430					
		1426					
		1421	↑		↑		
		1414				↓	↓
		1401					↑
		1398		—	—		↓
		1395		↑			
	Bending (δ, ρ, ω, τ) of OH of<br>primary & secondary alcohols	1385		↓	⤵ 1389	↓	↑
~1370 +/– 20		1374		⤴ 1372		↑	↑
		1368		↑			
		1363					
		1340		↓	⤵ 1343		↓
		1334				↑	
		1323		↑			
		1317	↑	↑		↑	⤵ 1319
	Amide III components of protein<br>1300 cm^{-1} α-helix	1312		↑			
~1310 – 1240	1285 & 1245 cm^{-1} random coil	1309				↑	
	1265 & 1235 cm^{-1} β-turn spectrum	1295		↓	—	↑	—
		1288	↑				
		1283				↑	
		1277		↑	↑		
		1268				—	—
		1251	⤴ 1248	⤴ 1250	⤴ 1247	⤵ 1256	⤴ 1250

(d) Important infrared absorption bands of carbohydrates

Peak Frequency (cm^{-1})	Assignment	Prominent adsorption peaks in control & stress experiments					
		Ctl.	MS1	MS2	OS1	OS2	OS3
1250 – 1220	υ_{as} P-O of >PO_2^- phosphodiester 1210 -1215 cm^{-1} for Z-form 1222 -1225 cm^{-1} for B-form 1240 -1250 cm^{-1} for A-form	1232		↓	↓		↑
		1225		↓	↓	↱ 1222	
		1218			↑		↑
		1214	↑	↑			
		1204				↓	↑
1240 – 1171	δ C-H of CH_2 coupled with C-O modes υ of C-O-C linkages - 1160: carbohydrate rich bands (Nichols, 1985)	1201			↑		
		1198	↑	↑			
		1187			↱ 1189		
		1179			↱ 1177		
		1169	↱ 1170	↱ 1167	↱ 1170	↱ 1172	↱ 1173
		1160				↑	↑
		1158	↑	↑	↑		
		1153				↑	↑
		1147	↓	↓			
		1137				↑	↑
		1127	↑				
1140 – 1070	υ_{as} C-O of saturated unbranched ethers in carbohydrates -	1124					
		1122		↑	↑		↑
		1115					
1300 – 1200/ 1050 - 1000	υ_{as} C-O of mixed (alkyl/ aryl) ethers in carbohydrates - 1240: carbohydrate rich bands (Nichols, 1985)	1110	↑		↑		
		1102		↑			↑
		1093			↑		
1200 – 1000	υ_s C-O, C-C, and C-O-H, C-O-C def of carbohydrates						

(d) Important infrared absorption bands of carbohydrates (cont.)

Peak Frequency (cm^{-1})	Assignment	Prominent adsorption peaks in control & stress experiments					
		Ctl.	MS1	MS2	OS1	OS2	OS3
1150 – 1070	υ_{as} C-O of secondary hydroxyl - 1150: carbohydrate of bacterial gum (Nichols, 1985)	1081	→ 1086		↑	↓	↑
		1077	↑				
		1068	→ 1067			↓	↓
1090 – 1085	υ_{as} P-O of $>PO_2^-$	1059	↑				
		1056			↑	↑	
1075 – 1000	υ_{as} C-O of primary hydroxyl - of carbohydrates	1044			↑		↑
		1032	↑	↑			
		1028				↓	↑
		1024		↑			
		1017	↑				
		1006			↑		

Supplemental Table 8: Characteristic key absorption frequencies in the mid-infrared spectral range from 1800 to 1000 cm^{-1} for Enterobacter-SAM under different types of stress. Refer to supplemental table 3 for additional information on adsorption peak assignment, and a table key.

(a) Important carbonyl (C=O) bands of lipids & fatty acids .

**Peak Frequency (cm^{-1})**	**Assignment**	**Prominent adsorption peaks in control & stress experiments**					
		**Ctl.**	**MS1**	**MS2**	**OS1**	**OS2**	**OS3**
1750 – 1700	υ carbonyl C=O	1749		↑ 1750	↑		
~1748 – 1740	υ C=O of ester (in PHA, confirmed by the presence of C-O of alkoxy group at 1311 cm^{-1}, and the CH=CH2 at 1027 cm^{-1} (Nwinyi et al., 2017))	1744				↑	↑
		1740		↑			
		1737	↑	↑ 1740			↑
		1736.8				↓	
~1734	υ C=O of saturated long-chain esters or ether esters	1736	↓	↓	↑ 1335		↓
		1730				↑	
~1722	υ C=O of saturated aldehydes	1721	↑				
~1715	υ C=O of saturated aliphatic ketones	1720	↓		↑	↓	↓
		1714				↑	
1713 - 1709	υ C=O of saturated aliphatic carboxylic acids	1702	↑				

(b) Important amide I and amide II bands of proteins

Peak Frequency (cm^{-1})	Assignment	Prominent adsorption peaks in control & stress experiments					
		Ctl.	MS1	MS2	OS1	OS2	OS3
		1696		↑		↑	
		1694			↑		
1715 – 1680	υ carbonyl C=O	1690	↑				
		1688.8					↑
~1690 – 1610	υC=O and υC-N of Amide I band components	1683				↓	
		1675	↑				
		1657.2		↓	↱ 1655	↰ 1658	↴ 1658
		1652					↑
		1639	↰ 1640	↰ 1640	↱ 1638.8	↰ 1640	↓
		1599.5				↑	
		1576			↑	↑	
		1570		↑			
		1561.5	↑				
		1546	↑	↰ 1548	↱ 1539	↳ 1545	↰ 1548
~1580 – 1470	υC-N, υ C-C, and δ N-H of Amide II band components	1540					↑
~1577<br>~1549		1537.6	— 1534.9	↰ 1541		↳ 1535	
~1538		1523	↑		↰ 1526		
~1518 - 1495		1514		↰	↑	↰	↑
~1520 – 1490	Aromatic rings (Incl. Tyr and Phe)	1501					

(c) Important complex infrared absorption bands of lipids, fatty acids and proteins

Peak Frequency (cm⁻¹)	Assignment	Prominent adsorption peaks in control & stress experiments					
		Ctl.	MS1	MS2	OS1	OS2	OS3
		1469	1473	1469.8	↑	↑	
~1475 – 1370		1459					↑
~1470	δC-H of CH_3, CH_2	1457	↑		↑		
~1456	C-H def of >CH_2	1454		1452		1453	↑
~1439		1442					↑
~1440 – 1395	C-O of carboxylic acid or -COO of carboxylate	1440	1438	↑	↓	↑	
		1422	1419	1419	1414	1419	1413.7
		1403	1401.7			↓	1404
		1390	1387	1391			
		1378		1373	1381	1376	
~1370 +/– 20	Bending (δ, ρ, ω, τ) of OH of primary & secondary alcohols (1370 cm-1 indicate carbohydrate-rich bands)	1370			1374		↑
		1357			↑		
		1353					↑
		1348	↑				
		1345		↑			
		1341					↑
		1338.7					
		1336	– 1334	1334	1335	↑	
		1331				↑	
		1325			↑		
		1319	1318				↑
		1312		1315	1311		
		1305	↑				↑
		1299	1291	1297	1297.8		
~1310 – 1240	Amide III components of protein 1300 cm⁻¹ α-helix 1285 & 1245 cm⁻¹ random coil 1265 & 1235 cm⁻¹ β-turn spectrum	1288.5					↑
		1280	1278		↓		
		1274				1273	↑
		1270.6		↑			
		1260			↑	↑	↑
		1253					
		1252					↑
		1246	1249	↑	↑	↑	

901

(d) Important infrared absorption bands of carbohydrates

Peak Frequency (cm^{-1})	Assignment	Prominent adsorption peaks in control & stress experiments					
		Ctl.	MS1	MS2	OS1	OS2	OS3
1250 – 1220	υ_{as} P-O of >PO_2^- phosphodiester	1234	1238	↑	1238	1230	1238
		1222		↓	↑	1219	1220.1
1240 – 1171	δ C-H of CH_2 coupled with C-O modes<br>υ of C-O-C linkages - 1160: carbohydrate rich bands (Nichols, 1985)	1209	↑		↑		
		1203		1206.6			1207
		1195	↑				
		1190				↑	↑
		1183.9	↑				
		1176				↑	↑
1200 – 1000	υ_s C-O, C-C, and C-O-H, C-O-C def of carbohydrates	1174	↑				
		1170				↑	
1300 – 1200/ 1050 - 1000	υ_{as} C-O of mixed (alkyl/ aryl) ethers in carbohydrates - 1240: carbohydrate rich bands (Nichols, 1985)	1164	1158	1163	1169	1160	1169
		1152		↑			

(d) Important infrared absorption bands of carbohydrates (cont.)

Peak Frequency (cm^{-1})	Assignment	Prominent adsorption peaks in control & stress experiments					
		Ctl.	MS1	MS2	OS1	OS2	OS3
		1148	↑				
		1143					↑
		1141				↓	
1150 – 1070	$υ_{as}$ C-O of secondary hydroxyl - 1150: carbohydrate of bacterial gum (Nichols, 1985)	1130	↑				
		1119	↓		1120	1121	1117
		1110			↑		
		1104		↑		1106	
		1098			↑		
1090 – 1085	$υ_{as}$ P-O of >PO_2^-	1091	1087	1090		↓	
		1083		↑			
		1077	1079	1080		1076	1082
		1070	1068	1069	1066		↓
		1063				1062	
		1055		↑			
1075 – 1000	$υ_{as}$ C-O of primary hydroxyl - of carbohydrates	1050				↑	↑
		1043	– 1041		1043.8		
		1039				1038	
		1033		↑			
		1022	1024	1020	1027	1020	1025
		1016			↑		↑
		1004					↑

Supplemental Table 9: Characteristic key absorption frequencies in the mid-infrared spectral range from 1800 to 1000 cm^{-1} for Enterobacter_HTF under different types of stress. Refer to supplemental table 4 for additional information on adsorption peak assignment, and a table key.

(a) Important carbonyl (C=O) bands of lipids & fatty acids .

Peak Frequency (cm^{-1})	Assignment	Prominent adsorption peaks in control & stress experiments					
		Ctl.	MS1	MS2	OS1	OS2	OS3
1750 – 1700	υ carbonyl C=O	1748			↑ 1745	↑ 1743	↑
~1748 – 1740	υ C=O of ester (in PHA, confirmed by the presence of C-O of alkoxy group at 1311 cm^{-1}, and the CH=CH2 at 1027 cm^{-1} (Nwinyi et al., 2017))	1735	↓ 1737	↓ 1740	↑	→ 1734	↑ 1736
~1734	υ C=O of saturated long-chain esters or ether esters	1722	↑				
~1722	υ C=O of saturated aldehydes	1721	→ 1717		↑		
~1715	υ C=O of saturated aliphatic ketones	1716	↓	↓	↑	↑	
1713 - 1709	υ C=O of saturated aliphatic carboxylic acids						

(b) Important amide I and amide II bands of proteins

Peak Frequency (cm^{-1})	Assignment	Prominent adsorption peaks in control & stress experiments					
		Ctl.	MS1	MS2	OS1	OS2	OS3
1715 – 1680	υ-carbonyl C=O	1696	1689.9	1691	1693.7	1695	1691
		1682				↑	
~1690 – 1610	υC=O and υC-N of Amide I band components	1678.7			1683		1680
		1667			↓		
		1657.7	1656.8	1658.3	↓	1656.6	1656
		1637.6	1639		1646	↓	1638.7
		1621			↑		
		1613			↑		
		1599			↑		
		1576		1578	1583		1573
		1570			↑		
~1580 – 1470	υC-N, υ C-C, and δ N-H of Amide II band components	1559			↑		
~1577		1552.2	↓	1553.9		↓	
~1549<br>~1538		1546	1544	1541	↓	↓	↓
~1518 - 1495		1539					↑
~1520 – 1490	Aromatic rings (Incl. Tyr and Phe)	1537	↑				
		1536.6				↑	
		1534			↑		
		1517	1515	— 1520	— 1523	1516	1515
		1505			↑		

(c) Important complex infrared absorption bands of lipids, fatty acids and proteins

Peak Frequency (cm^{-1})	Assignment	Prominent adsorption peaks in control & stress experiments					
		**Ctl.**	**MS1**	**MS2**	**OS1**	**OS2**	**OS3**
		1468	1470	↑	↑	↓	↓
		1464			↑		
~1475 – 1370		1454	1455	1455	1443	1455	↑
~1470<br>~1456	δC-H of CH_3, CH_2	1437.5	1441	1441	1440	1440	1437
~1439	C-H def of $>CH_2$	1434			↓		
~1440 – 1395	C-O of carboxylic acid or	1420				↓	
	-COO of carboxylate	1418			↑		↓
		1414		↓			
		1402	↓	1398	↓		↓
		1393					↑
	Bending (δ, ρ, ω, τ) of OH of	1385	1390	1386.9		1392	1383
~1370 +/– 20	primary & secondary alcohols<br>(1370 cm-1 indicate	1379				↑	
	carbohydrate-rich bands)	1364.4	1370	1373	1358	↑<br>– 1367	1369
		1349				↑	
		1346	↑				
		1344			↑		
		1335	1331	1340	1331	↑	1340
		1322		↑		↑	
		1318					↑
		1315			↑		
		1312				↑	
		1302	↑	↓			↑
	Amide III components of protein<br>1300 cm^{-1} α-helix	1292				– 1297	
~1310 – 1240	1285 & 1245 cm^{-1} random coil	1286.9	1281			1279	1288
	1265 & 1235 cm^{-1} β-turn spectrum	1268	↑		↑		
		1258.1		1259		1260	1264
		1251			↑	↓	
		1248		↑		↑	
		1243	↑				

951

(d) Important infrared absorption bands of carbohydrates region

Peak Frequency (cm^{-1})	Assignment	Prominent adsorption peaks in control & stress experiments					
		Ctl.	MS1	MS2	OS1	OS2	OS3
1250 – 1220	υ_{as} P-O of $>PO_2^-$ phosphodiester	1236		↑	↑	↑	↑
		1227.7	1225	1226	1221	1229	– 1224
		1216	1213	1213			
1240 – 1171	δ C-H of CH_2 coupled with C-O modes<br>υ of C-O-C linkages - 1160: carbohydrate rich bands	1193		1190	1197		
		1183				↑	
		1172.7	– 1176	1172	1175	↓	↓
1200 – 1000	υ_s C-O, C-C, and C-O-H, C-O-C def of carbohydrates	1159.5	1163	1154	1155	1153	1166
1300 – 1200/ 1050 - 1000	υ_{as} C-O of mixed (alkyl/ aryl) ethers in carbohydrates - 1240: carbohydrate rich bands						

(d) Important infrared absorption bands of carbohydrates (cont.)

Peak Frequency (cm^{-1})	Assignment	Prominent adsorption peaks in control & stress experiments					
		Ctl.	MS1	MS2	OS1	OS2	OS3
		1142.5		1138			1150
		1136					↑
		1133			↓		
1150 – 1070	$υ_{as}$ C-O of secondary hydroxyl - 1150: carbohydrate of bacterial gum (Nichols, 1985)	1128			1126	↑	
		1114	↑	1110	1109	1117	1118.9
		1098.6	1101	↓		1098	1106
1090 – 1085	$υ_{as}$ P-O of >PO_2^-	1084	1082	1085		1081	1082
		1078		↑	↑		
		1072	↑				
		1069					↑
		1065		1061		1061	
		1059	↑				
		1053			1051	1051	1056
1075 – 1000	$υ_{as}$ C-O of primary hydroxyl - of carbohydrates	1038.9	1034		1034	1031	
		1026	1018	—	1022	1016	
		1017		↑			
		1011			1008		1009

Supplemental Table 10: Characteristic key absorption frequencies in the mid-infrared spectral range from 1800 to 1000 cm^{-1} for Sphingomonas-SAM under different types of stress. Refer to supplemental table 4 for additional information on adsorption peak assignment, and a table key.

(a) Important carbonyl (C=O) bands of lipids & fatty acids

Peak Frequency (cm^{-1})	Assignment	Prominent adsorption peaks in control & stress experiments					
		Ctl.	MS1	MS2	OS1	OS2	OS3
1750 – 1700	υ carbonyl C=O	1740			↓		↓
~1748 – 1740	υ C=O of ester	1737	↑ 1738	↓ 1737		↓ 1731	↓ 1739
~1734	υ C=O of saturated long-chain esters or ether esters	1728		↑			
~1722	υ C=O of saturated aldehydes	1720	↓	↑	↑ 1715	↑ 1717	↑
~1715	υ C=O of saturated aliphatic ketones	1714					↑
1713 - 1709	υ C=O of saturated aliphatic carboxylic acids						

(b) Important amide I and amide II bands of proteins

Peak Frequency (cm^{-1})	Assignment	Prominent adsorption peaks in control & stress experiments					
		Ctl.	MS1	MS2	OS1	OS2	OS3
		1702					
1715 – 1680	υ-carbonyl C=O	1692	↑		↑	↑	↑
		1682	↓		↓		
		1675				↑	
		1658.4	1658	1660	1653	1659.2	1657
~1690 – 1610	υC=O and υC-N of Amide I band components	1645.3		1650		1647	
		1635.8	1636.4	1639	↓	1632	1639.4
		1610				↑	
		1587				↑	
		1570		– 1569.7		1575	
~1580 – 1470	υC-N, υ C-C, and δ N-H of Amide II band components	1557	↓	1552	1554	1554	
~1577		1545.5	1544	1539	1546	1546	1549.2
~1549		1535				↓	↓
~1538		1525	↓				
~1518 - 1495		1520				↓	
~1520 – 1490	Aromatic rings (Incl. Tyr and Phe)	1516					↓
		1511	↓				
		1507				↓	
		1502			↓		
		1492.6	1493			1488	

(c) Important complex infrared absorption bands of lipids, fatty acids and proteins

Peak Frequency (cm^{-1})	Assignment	Prominent adsorption peaks in control & stress experiments					
		**Ctl.**	**MS1**	**MS2**	**OS1**	**OS2**	**OS3**
		1470	1469	—	1467	1471	1468
~1475 – 1370		1457		↑		↑	
~1470	δC-H of CH_3, CH_2	1456.9	—		1449.7		1455
~1456	C-H def of >CH_2	1447		↑			↑
~1439<br>~1440 – 1395	C-O of carboxylic acid or -COO of carboxylate	1438.3	↓	1437	↓	1434	1436
		1419	↓	1415	1414	1421	1416
		1404.9	1406			1411	1400
		1393.2	1390	1392	1397	1399	1390
		1388	1379		1382	1387.3	
~1370 +/– 20	Bending (δ, ρ, ω, τ) of OH of primary & secondary alcohols (1370 cm-1 indicate carbohydrate-rich bands)	1378		↑			
		1369	1367	1368	↓	1365.8	1371
		1352.3			↑	1349.5	
		1346	↓	1347	1340	1340	
		1340					↑
		1333	↓	1329		1327	
		1324			1323	1323	
		1318			↓		↑
		1313		↑			
		1310	↑				
		1302			↓	1306	1300
		1297			↓		
		1292	↓			↑	
		1286		↑			
~1310 – 1240	Amide III components of protein<br>1300 cm^{-1} α-helix<br>1285 & 1245 cm^{-1} random coil<br>1265 & 1235 cm^{-1} β-turn spectrum	1280			↑	↑	
		1277		1276			
		1268		1260	1267		1265
		1256				↑	↑
		1247		↑	1253	1240	

1009

(d) Important infrared absorption bands of carbohydrates

**Peak Frequency (cm^{-1})**	**Assignment**	**Prominent adsorption peaks in control & stress experiments**					
		**Ctl.**	**MS1**	**MS2**	**OS1**	**OS2**	**OS3**
1250 – 1220	υ_{as} P-O of >PO_2^- phosphodiester	1237	1233	1238	1239		
		1229		1227	↑		
		1216		↑	1218	1213	1219
1240 – 1171	δ C-H of CH_2 coupled with C-O modes	1210	↑				
	υ of C-O-C linkages - 1160: carbohydrate rich bands	1204	1200				
		1192	1187.8	↓	1189	1191	
		1172	1171	1175.2	1176	1179	1175
		1161	1157.6	1162	1158	1160	
		1145	↑	↑		↑	↑
1140 – 1070	υ_{as} C-O of saturated unbranched ethers in carbohydrates -	1136				↑	
		1132	↑				↑
1200 – 1000	υ_s C-O, C-C, and C-O-H, C-O-C def of carbohydrates						
1300 – 1200/ 1050 - 1000	υ_{as} C-O of mixed (alkyl/ aryl) ethers in carbohydrates - 1240: carbohydrate rich bands						

(d) Important infrared absorption bands of carbohydrates (cont.)

**Peak Frequency (cm^{-1})**	**Assignment**	**Prominent adsorption peaks in control & stress experiments**					
		**Ctl.**	**MS1**	**MS2**	**OS1**	**OS2**	**OS3**
		1128				↑	
		1124		↑	↑		
		1120					↑
1150 – 1070	υ_{as} C-O of secondary hydroxyl - 1150: carbohydrate of bacterial gum (Nichols, 1985)	1114					
		1107.2			1102.9	1109	
		1094.5		1089		1091	
1090 – 1085	υ_{as} P-O of $>PO_2^-$	1082	1079	1079	1085	1081	↑
		1079		1065	1069		
		1059	1061	1056	1053	1053	
		1044	1046	1038	1038	1041	
1075 – 1000	υ_{as} C-O of primary hydroxyl - of carbohydrates	1033	1034		1032	1027	1030
		1027	↑				
		1018			1022		
		1006	1008	1008	1010	1009	1011

Supplemental Table 11: Characteristic key absorption frequencies in the mid-infrared spectral range from 1800 to 1000 cm^{-1} for Sphingomonas-HTF under different types of stress. Refer to supplemental table 4 for additional information on adsorption peak assignment, and a table key.

(a) Important carbonyl (C=O) bands of lipids & fatty acids

Peak Frequency (cm^{-1})	Assignment	Prominent adsorption peaks in control & stress experiments					
		Ctl.	MS1	MS2	OS1	OS2	OS3
1750 – 1700	υ carbonyl C=O	1749.2		↑			
~1748 – 1740	υ C=O of ester	1747			↓		
~1734	υ C=O of saturated long-chain esters or ether esters	1742		↱ 1740	↰ 1748	↴ 1746	—
~1722	υ C=O of saturated aldehydes	1738			↑		↑
~1715	υ C=O of saturated aliphatic ketones	1728	↓	— 1727	↓	↓	↱ 1727
1713 - 1709	υ C=O of saturated aliphatic carboxylic acids	1720					↑

(b) Important amide I and amide II bands of proteins

Peak Frequency (cm^{-1})	Assignment	Prominent adsorption peaks in control & stress experiments					
		Ctl.	MS1	MS2	OS1	OS2	OS3
		1691				↑	
1715 – 1680	υ-carbonyl C=O	1688	↑	↓			
		1682				1680	
		1678					↑
		1675		↑			
~1690 – 1610	υC=O and υC-N of Amide I band components	1659		1660	1658	1658	1658
		1652	↓				
		1640	1639	1641	1639	1638	1641
		1600		↑			
		1584		↑			
		1573		↑			
~1580 – 1470	υC-N, υ C-C, and δ N-H of Amide II band components	1551		↑			
~1577							
~1549		1543.3	1542.5	1539	1549.4	1549	1546
~1538		1526					↑
~1518 - 1495		1516.6	↓	1522	1515	↓	1514
~1520 – 1490	Aromatic rings (Incl. Tyr and Phe)	1501					↑
		1497		↑			
		1495	↑				

(c) Important complex infrared absorption bands of lipids, fatty acids and proteins

Peak Frequency (cm^{-1})	Assignment	Prominent adsorption peaks in control & stress experiments					
		Ctl.	MS1	MS2	OS1	OS2	OS3
		1469	—	↓	↓	↓	↓ 1471
~1475 – 1370		1456	↓	1455	1453	1454	
~1470	δC-H of CH_3, CH_2						
~1456	C-H def of >CH_2	1439	↑	1438	1443	1442	1443
~1439		1430					↑
~1440 – 1395	C-O of carboxylic acid or -COO of carboxylate	1424		↑	↑		
		1413	↓	1409	↓	—	1407
		1400			↑		
		1393	↑	1397	1389.8	1395	
		1383	↑	↑		1387	
~1370 +/– 20	Bending (δ, ρ, ω, τ) of OH of primary & secondary alcohols (1370 cm-1 indicate carbohydrate-rich bands)	1370	1369	↑	↓	1375	1376
		1361	↑				
		1357		↑			
		1351			1347		1357
		1337	↓	1332	↓	1336	1345
		1325	↑	↑	↓	↑	
		1319					↑
		1307	↑	1302	1306	1312	↓
		1299				↑	
~1310 – 1240	Amide III components of protein 1300 cm^{-1} α-helix 1285 & 1245 cm^{-1} random coil 1265 & 1235 cm^{-1} β-turn spectrum	1288	↑	1294	1297	1284	1295
		1275					↑
		1268		↑	↑		
		1264	↑				
		1261					↑
		1258			↑		
		1249	↑	1255	— 1246	1255	1252

(d) Important infrared absorption bands of carbohydrates

Peak Frequency (cm^{-1})	Assignment	Prominent adsorption peaks in control & stress experiments					
		Ctl.	MS1	MS2	OS1	OS2	OS3
1250 – 1220	υ_{as} P-O of $>PO_2^-$ phosphodiester	1235	↓	↓ 1244		↓ 1244	↑ 1236
		1225	↓	↓ 1233	↓	↓ 1228	↑ 1227
	δ C-H of CH_2 coupled with C-O modes	1212	↑ 1213	↑ 1210			↓ 1215
1240 – 1171	υ of C-O-C linkages - 1160: carbohydrate rich bands (Nichols, 1985)	1194	↑ 1192	↑ 1198			↑ 1197
		1184					↑
		1171	—	↑			
		1160	↑		↓ 1166	↓ 1166	↑ 1169
		1151	↓	↑ 1148	↑ 1154	↓ 1155	
		1137					↑
		1134			↑		
1150 – 1070	υ_{as} C-O of secondary hydroxyl - 1150: carbohydrate of bacterial gum	1122	↑ 1123		↑	↑ 1126	↑ 1123
		1114		↑			
1140 – 1070	υ_{as} C-O of saturated unbranched ethers in carbohydrates -	1107	↑				↓ 1104
		1101		↑			
1090 – 1085	υ_{as} P-O of $>PO_2^-$	1088.5	↓				↑ 1091
		1081	↓	↑ 1083	↑ 1084	↑ 1082	
		1076					↑
		1070	↑ 1071				
1075 – 1000	υ_{as} C-O of primary hydroxyl - of carbohydrates	1060	↑ 1061	↑ 1063			↑ 1057
		1056		↑			
		1040	↑	↑			↑ 1043
1200 – 1000	υ_s C-O, C-C, and C-O-H, C-O-C def of carbohydrates	1030	↓	↑ 1032	↑	↑ 1032	
		1020	↓			↓ 1024	↑ 1024
1300 – 1200/ 1050 - 1000	υ_{as} C-O of mixed (alkyl/ aryl) ethers in carbohydrates - 1240: carbohydrate rich bands	1015		↑			
		1005					↑

References

1. Nunes Da Rocha U, Cadillo-Quiroz H, Karaoz U, Rajeev L, Klitgord N, Dunn S, et al. Isolation of a significant fraction of non-phototroph diversity from a desert Biological Soil Crust. *Front Microbiol* 2015; **6**.
2. Lane DJ, Pace B, Olsen GJ, STAHLt DA, SOGINt ML, Pace NR. Rapid determination of 16S ribosomal RNA sequences for phylogenetic analyses. *Proc Natl Acad Sci USA* 1985.
3. Rochelle PA, Fry JC, Parkes RJ, Weightman AJ. DNA extraction for 16S rRNA gene analysis to determine genetic diversity in deep sediment communities. *FEMS Microbiol Lett* 1992; **100**: 59–65.
4. Letunic I, Bork P. Interactive Tree Of Life (iTOL) v5: an online tool for phylogenetic tree display and annotation. *Nucleic Acids Res* 2021; **49**: W293–W296.
